# Natural carboxyterminal truncation of human CXCL10 attenuates glycosaminoglycan binding, CXCR3A signaling and lymphocyte chemotaxis, while retaining angiostatic activity

**DOI:** 10.1186/s12964-023-01453-1

**Published:** 2024-02-02

**Authors:** Luna Dillemans, Karen Yu, Alexandra De Zutter, Sam Noppen, Mieke Gouwy, Nele Berghmans, Lisa Verhallen, Mirre De Bondt, Lotte Vanbrabant, Stef Brusselmans, Erik Martens, Dominique Schols, Patrick Verschueren, Mette M. Rosenkilde, Pedro Elias Marques, Sofie Struyf, Paul Proost

**Affiliations:** 1 Laboratory of Molecular Immunology, Department of Microbiology, Immunology and Transplantation, Rega Institute, KU Leuven, Leuven, Belgium; 2 Laboratory of Virology and Chemotherapy, Department of Microbiology, Immunology and Transplantation, Rega Institute, KU Leuven, Herestraat 49 Box 1042, Leuven, Belgium; 3 Laboratory of Molecular Pharmacology, Department of Biomedical Sciences, Faculty of Health and Medical Sciences, University of Copenhagen, 2200 Copenhagen, Denmark; 4 Laboratory of Immunobiology, Department of Microbiology, Immunology and Transplantation, Rega Institute, KU Leuven, Leuven, Belgium; 5 Skeletal Biology and Engineering Research Center, Department of Development and Regeneration, KU Leuven, Leuven, Belgium

**Keywords:** Angiogenesis, Chemokine, CXCL10, Lymphocytes, Posttranslational modifications, Proteolysis, Solid phase peptide synthesis

## Abstract

**Background:**

Interferon-γ-inducible protein of 10 kDa (IP-10/CXCL10) is a dual-function CXC chemokine that coordinates chemotaxis of activated T cells and natural killer (NK) cells via interaction with its G protein-coupled receptor (GPCR), CXC chemokine receptor 3 (CXCR3). As a consequence of natural posttranslational modifications, human CXCL10 exhibits a high degree of structural and functional heterogeneity. However, the biological effect of natural posttranslational processing of CXCL10 at the carboxy (C)-terminus has remained partially elusive. We studied CXCL10_(1–73)_, lacking the four endmost C-terminal amino acids, which was previously identified in supernatant of cultured human fibroblasts and keratinocytes.

**Methods:**

Relative levels of CXCL10_(1–73)_ and intact CXCL10_(1–77)_ were determined in synovial fluids of patients with rheumatoid arthritis (RA) through tandem mass spectrometry. The production of CXCL10_(1–73)_ was optimized through Fmoc-based solid phase peptide synthesis (SPPS) and a strategy to efficiently generate human CXCL10 proteoforms was introduced. CXCL10_(1–73)_ was compared to intact CXCL10_(1–77)_ using surface plasmon resonance for glycosaminoglycan (GAG) binding affinity, assays for cell migration, second messenger signaling downstream of CXCR3, and flow cytometry of CHO cells and primary human T lymphocytes and endothelial cells. Leukocyte recruitment in vivo upon intraperitoneal injection of CXCL10_(1–73)_ was also evaluated.

**Results:**

Natural CXCL10_(1–73)_ was more abundantly present compared to intact CXCL10_(1–77)_ in synovial fluids of patients with RA. CXCL10_(1–73)_ had diminished affinity for GAG including heparin, heparan sulfate and chondroitin sulfate A. Moreover, CXCL10_(1–73)_ exhibited an attenuated capacity to induce CXCR3A-mediated signaling, as evidenced in calcium mobilization assays and through quantification of phosphorylated extracellular signal-regulated kinase-1/2 (ERK1/2) and protein kinase B/Akt. Furthermore, CXCL10_(1–73)_ incited significantly less primary human T lymphocyte chemotaxis in vitro and peritoneal ingress of CXCR3^+^ T lymphocytes in mice. In contrast, loss of the four endmost C-terminal residues did not affect the inhibitory properties of CXCL10 on migration, proliferation, wound closure, phosphorylation of ERK1/2, and sprouting of human microvascular endothelial cells.

**Conclusion:**

Our study shows that the C-terminal residues Lys^74^-Pro^77^ of CXCL10 are important for GAG binding, signaling through CXCR3A, T lymphocyte chemotaxis, but dispensable for angiostasis.

**Graphical Abstract:**

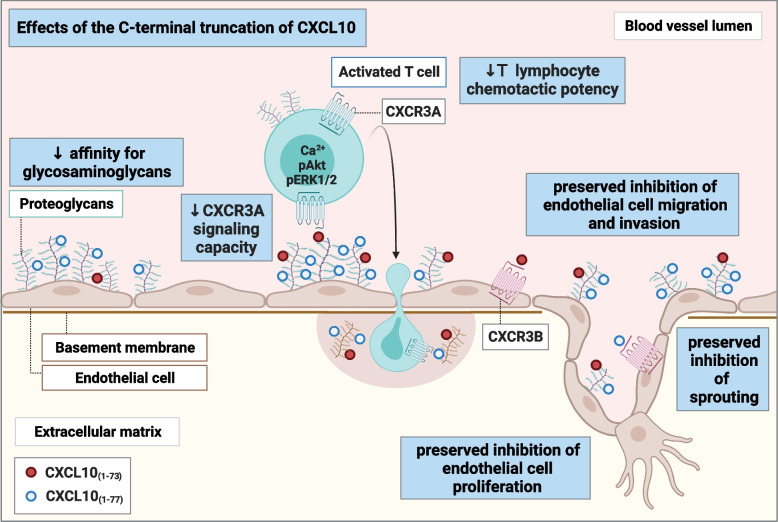

**Supplementary Information:**

The online version contains supplementary material available at 10.1186/s12964-023-01453-1.

## Background

The superfamily of chemotactic cytokines or chemokines encompasses structurally similar, low molecular mass (7-12 kDa) proteins that govern directional leukocyte trafficking through interaction with chemokine-type G protein-coupled receptors (GPCRs) and glycosaminoglycans (GAGs) [1–4]. From a biochemical perspective, chemokines may be subdivided into four major subfamilies based on the number and positioning of the N-terminally located conserved cysteine residues [5–7]. CXC chemokines contain one random amino acid (‘X’) in between these cysteines and are further subcategorized based on the presence or absence of a Glu-Leu-Arg (‘ELR’) sequence located anterior of the CXC motif [6]. ELR^+^ CXC chemokines bind CXCR1 and/or CXCR2 and chemo-attract neutrophils, whereas ELR^−^ CXC chemokines primarily mediate directional lymphocyte trafficking [1, 7, 8]. Chemokines can also be categorized into functional subclasses, separating those with inflammatory as opposed to homeostatic actions, whereby ‘dual-function’ chemokines exhibit activities reminiscent of both inflammation and homeostasis [9, 10]. Interferon-γ-inducible protein of 10 kDa (IP-10/CXCL10) is a dual-function ELR^−^ CXC chemokine that coordinates chemotaxis of activated CD4^+^ T_H_1 cells, CD8^+^ T cells, natural killer (NK) cells and NKT cells via interaction with its GPCR, CXC chemokine receptor 3 (CXCR3) [11–18]. In addition, CXCL10 exhibits anti-angiogenic activity [19–21].

Posttranslational modifications (PTMs) have been acknowledged as mechanisms that regulate chemokine functioning in vitro and in vivo through modulating the affinity and selectivity for GPCRs and GAGs [22–24]. PTMs are executed by specific enzymes that are co-expressed during inflammation [23]. These modifications may include proteolytic truncation, glycosylation, nitration and citrullination [23]. CXCL10 is no exception to this rule and is highly susceptible to site-specific N- and C-terminal proteolytic processing [25, 26]. In addition to intact CXCL10_(1–77),_ purification of natural CXCL10 from cell culture supernatant of stimulated human fibroblasts, primary keratinocytes, MG-63 osteosarcoma cells, human umbilical cord endothelial cells, and peripheral blood mononuclear cells (PBMC) revealed multiple natural CXCL10 proteoforms [27–33]. These natural isoforms of CXCL10 were missing four C-terminal amino acids (Lys^74^, Arg^75^, Ser^76^, and Pro^77^), lacking two to five N-terminal residues (Val^1^, Pro^2^, Leu^3^, Ser^4^, and Arg^5^) and/or containing a citrulline instead of Arg^5^ [27–31]. N-terminal truncation of CXCL10 by the enzyme dipeptidylpeptidase IV (DPPIV/CD26) generates CXCL10_(3–77)_, which functions as a chemotaxis antagonist with retained angiostatic properties [34]. The antagonistic activities of CXCL10_(3–77)_ in terms of lymphocyte chemotaxis were also demonstrated in vivo [35, 36]*.* CD26 inhibition in C57BL/6 mice through sitagliptin administration restored lymphocyte-attracting activity of intraperitoneally injected human CXCL10, and of endogenous murine CXCL10 (mCXCL10), resulting in enhanced recruitment of CXCR3^+^ lymphocytes towards the peritoneal cavity and B16F10 melanoma tumors, respectively [35, 36]. In addition, natural N-terminally truncated CXCL10_(3–77)_ was detected in plasma of patients with hepatitis C virus (HCV) infection and in urine of patients with bladder carcinoma and active tuberculosis [37–42]. Hence, these findings provide evidence that PTMs of CXCL10 have in vivo biological significance in clinical settings.

In contrast to N-terminal proteolysis, the natural C-terminal truncation of human CXCL10 has been explored to a limited extent, despite its verified presence in natural conditioned media of human cells [27, 29, 30]. The C-terminal residues were implicated in the anti-angiogenic and anti-parasitic properties of CXCL10 [43, 44]. A C-terminal fragment of CXCL10, spanning the α-helical and coiled domain residues Pro^56^-Pro^77^, was reported to inhibit in vitro vascular endothelial growth factor (VEGF)-induced endothelial cell migration and in vivo vessel formation to a comparable extent as intact CXCL10_(1–77)_ [43]. Moreover, virulence factor glycoprotein-63 (GP63) of *Leishmania Major* cleaves off the C-terminal α-helix of CXCL10 at Ala^60^-Lys^62^, generating a CXCL10 proteoform with attenuated T cell chemotactic potential in vitro [44]. In terms of structural modeling, C-terminal residues Ser^76^-Pro^77^ of human CXCL10 were not successfully modeled via NMR spectroscopy and crystallography [45, 46]. Therefore, prediction of the precise location and proximity of the C-terminal amino acids relative to other residues in the peptide backbone of CXCL10 is still speculative, making structure–activity predictions challenging. C-terminal residues of mCXCL10 have been investigated to a more elaborate extent and were implicated in GAG and receptor binding [47–50]. Similar to mCXCL10, the four shedded amino acids in C-terminally truncated human CXCL10_(1–73)_ consist of two positively charged basic amino acids (Lys^74^ and Arg^75^), which also hints towards potentially diminished GAG affinity. Hence, these intriguing findings suggest that C-terminal residues may profoundly shape the functions of human CXCL10. This sparked our interest to perform an in-depth characterization of hallmark chemokine properties of C-terminally truncated human CXCL10_(1–73)_.

The aim of the present study was to evaluate the effects of the naturally occurring C-terminal truncation of CXCL10 on the functional properties of this chemokine. We report on the identification of natural CXCL10_(1–73)_ in synovial fluids of patients with rheumatoid arthritis (RA) and discovered that the concentration of CXCL10_(1–73)_ was higher compared to that of intact CXCL10_(1–77)_. To study CXCL10_(1–73)_ biology, we introduced a strategy for Fmoc-based solid phase peptide synthesis (SPPS). We discovered that the C-terminal truncation of CXCL10 attenuated the interaction with GAGs, the signaling properties through CXCR3A, and the ability to attract T lymphocytes in vitro and in vivo. However, the angiostatic properties of CXCL10, including the inhibition of migration, proliferation, wound closure, phosphorylation of extracellular signal-regulated kinase-1/2 (ERK1/2), and sprouting of endothelial cells, were not affected by the C-terminal processing.

## Results

### Identification and chemical synthesis of CXCL10_(1–73)_

**Fig. 1:**
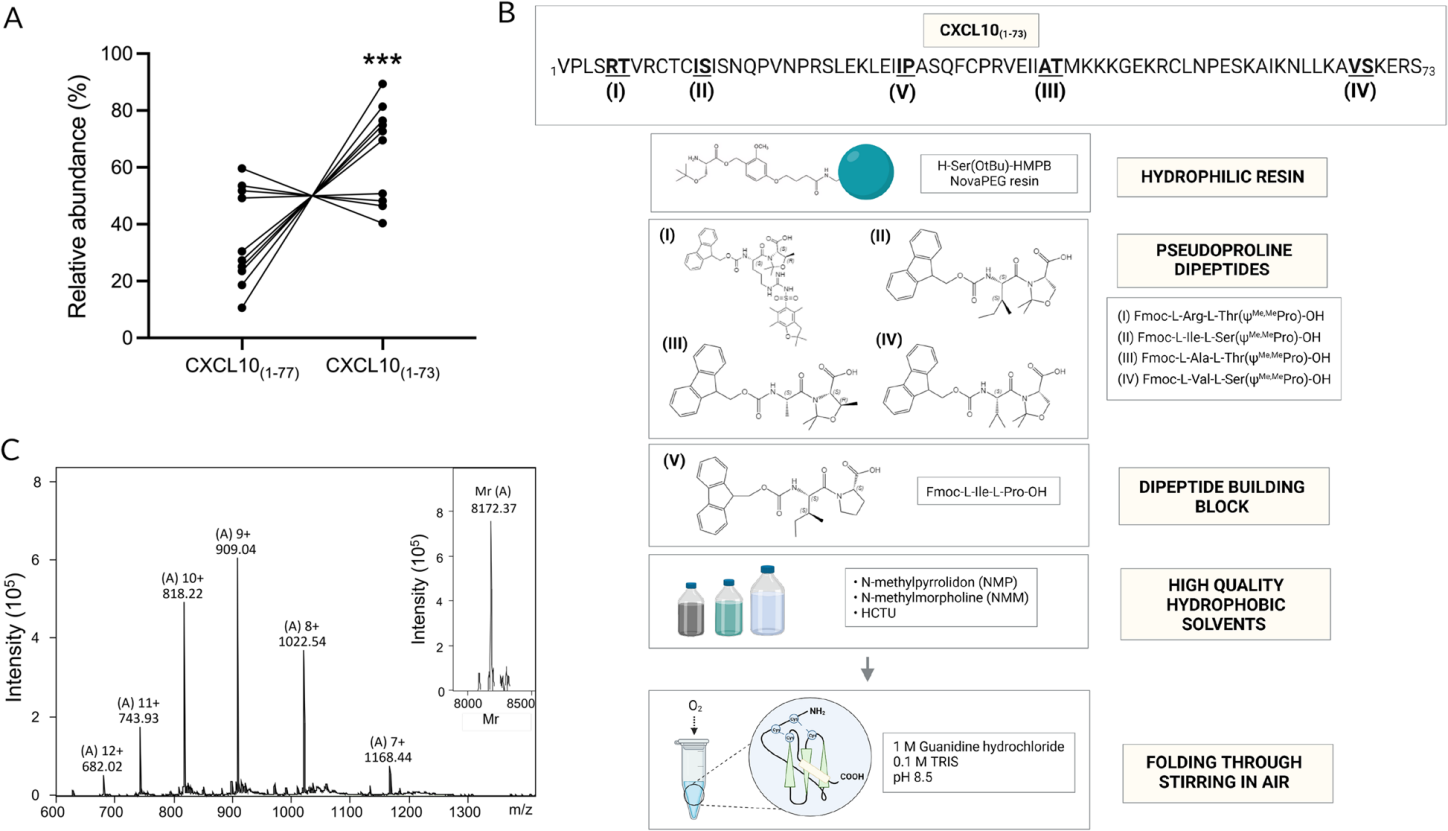
Identification of natural CXCL10_(1–73)_ and chemical synthesis of CXCL10_(1–73)_. **A** Detection of relative levels of CXCL10_(1–77)_ and CXCL10_(1–73)_ by top down-tandem mass spectrometry indicates upregulation of CXCL10_(1–73)_ in the synovial fluids of patients with RA. Data are shown as paired dots for each patient (*n* = 10). Relative levels of CXCL10_(1–77)_ and CXCL10_(1–73)_ (ratio of the intensity of the respective proteoform to the sum of intensities of both proteoforms) were compared using an unpaired t-test (*** *p* ≤ 0.001). **B** Four crucial aspects were defined to ensure a successful SPPS using Fmoc chemistry including the combined use of a hydrophilic resin, pseudoproline dipeptides and a dipeptide building block (indicated I-IV) at crucial positions and the continuous application of high quality solvents. The amino acid sequence of CXCL10_(1–73)_ is depicted with the key positions where pseudoproline dipeptides and the dipeptide building block (bold and underlined) were incorporated. **C** The intensity of the detected ions with their respective mass/charge (m/z) ratio are displayed. The relative molecular mass (Mr) of CXCL10_(1–73)_ was calculated with Bruker deconvolution software (inset on the right) based on the detected ions, i.e., the ions marked by [A] with 7 to 12 positive charges. The experimental Mr (8172.37) corresponded to the calculated theoretical Mr (8173.65)

C-terminally truncated CXCL10 proteoforms were previously demonstrated to be produced by IFN-γ-stimulated human skin/muscle-derived fibroblasts in vitro [27]. Although immunoassays fail to discriminate between CXCL10 proteoforms, total CXCL10 was found to be highly upregulated in the synovial fluid of patients with RA [51] and synovial fibroblasts were identified as major producers of this chemokine [52, 53]. Therefore, we investigated the relative presence of natural CXCL10_(1–73)_ compared to unprocessed and fully active CXCL10 in synovial fluid samples of patients with RA. Although heterogeneity in the relative presence of CXCL10_(1–73)_ and CXCL10_(1–77)_ was detected in our cohort of RA patients, we found that mean concentrations of CXCL10_(1–73)_ were relatively increased compared to CXCL10_(1–77)_ (Fig. 1A). To characterize the biological properties of natural C-terminally truncated CXCL10_(1–73)_, this chemokine was chemically synthetized. Initial SPPS was performed using standard reagents for chemokine synthesis [54, 55]. When an Fmoc-Ser(But)-Wang resin was used with 2-(1H-7-azabenzotriazol-1-yl)-1,1,3,3-tetramethyluronium hexafluorophosphate (HATU) and di-isopropylethylamine (DIEA) as a coupling system, a remarkably poor yield of correctly synthesized CXCL10_(1–73)_ (± 0.026%) was obtained (theoretical relative molecular mass [Mr] 8173.65, experimental Mr 8172.72) (Fig. S1A). Since the aforementioned coupling reagents have been generally acknowledged to result in highly efficient coupling [56], we assumed that the synthesis failure may have been caused by the nature of the amino acid sequence of the protein itself. Hence, in the sequence of CXCL10_(1–73)_, 48% of the amino-acids were found to have hydrophobic side-chains. To prevent synthesis failure due to hydrophobicity, pseudoprolines were incorporated at key positions based on both theoretically predicted and experimentally determined challenging regions that required multiple deprotection steps during the failed synthesis [57]. Hence, pseudoproline dipeptides were inserted at Arg^5^-Thr^6^, Ile^12^-Ser^13^, Ala^43^-Thr^44^ and Val^68^-Ser^69^ in the peptide backbone of CXCL10*.* In addition, 1,1,3,3-tetramethyluronium hexafluorophosphate (HCTU) and 4-methylmorpholine (NMM) were used as alternative high quality coupling reagents [58–60]. Despite these modifications in the SPPS,  solely a limited amount of CXCL10_(1–73)_ was successfully synthesized. A major contaminant was detected, i.e., N-terminally shortened acetylated CXCL10_(31–73)_ (theoretical Mr 4870.78, experimental Mr 4868.86) (Fig. S1B). The acetylation clearly points towards a synthesis artefact, resulting from impaired amide bond formation between Ile^30^ and Pro^31^. Coupling of amino acids to C-terminal resin-attached Pro residues is often more challenging given the reduced reactivity of the secondary amine located in the proline pyrrolidine ring structure. The formation of this shortened peptide was circumvented via the selective incorporation of a specific dipeptide building block at Ile^30^-Pro^31^, i.e., Fmoc-L-Ile-L-Pro (Fig. S1C). Subsequently, the successfully obtained purified crude linear material was folded, as CXCL10 contains two disulfide bridges (Cys^9^-Cys^36^ and Cys^11^-Cys^53^). Initial folding was performed through incubation in 150 nM tris(hydroxymethyl)-aminomethane (Tris; pH 8.6) supplemented with 3 mM ethylenediaminetetraacetic acid (EDTA), 0.3 mM reduced glutathione (GSH) 3 mM oxidized glutathione (GSSG), and 1 M guanidine hydrochloride for 5 h under continuous rotation [54]. However, since two glutathione residues (each with Mr 307.33) remained covalently coupled to the cysteines in the peptide backbone after the folding procedure (theoretical Mr 8788.31, experimental Mr 8785.02) (Fig. S1D), this approach resulted in a significant portion of the synthetic protein being incompletely folded. Therefore, an alternative folding methodology was applied, in which the crude linear protein was incubated in 1.0 M guanidine hydrochloride and 0.1 M Tris (pH 8.5) whilst continuously stirred in air for 24 h to allow formation of the disulfide bridges [61]. Using this strategy of concomitant use of a hydrophilic resin, pseudoproline dipeptides, a dipeptide building block at pivotal positions, high quality hydrophobic solvents and oxidative folding under exposure of air (Fig. 1B), correctly synthesized and folded CXCL10_(1–73)_ was obtained (Fig. 1C; theoretical Mr of the folded protein 8173.65, experimentally determined Mr 8172.37).

### CXCL10_(1–73)_ has reduced affinity for GAGs compared to native CXCL10_(1–77)_

**Fig. 2:**
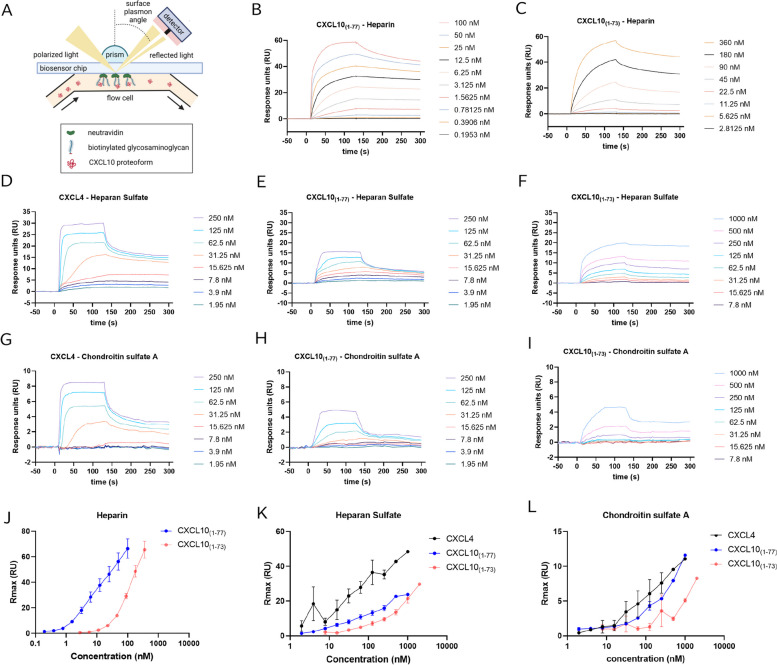
Glycosaminoglycan affinity of C-terminally truncated CXCL10_(1–73)_ is reduced compared to intact CXCL10_(1–77)_. CXCL10_(1–77)_ and CXCL10_(1–73)_ at varying concentrations were sent over the neutravidin-coated CM4 Biosensor chip surface on which biotinylated heparin, HS or CS-A were immobilized. **A** A schematic illustration of the experimental set-up is shown. Representative SPR sensorgrams are shown (from 4 independent experiments) displaying the affinity for **B**, **C** heparin, **D**-**F** HS and **G**-**I** CS-A of **B**, **E**, **H** CXCL10_(1–77)_, **C**, **F**, **I** CXCL10_(1–73)_ and **D**, **G** CXCL4. SPR sensorgrams were obtained after subtracting the baseline signal of the reference channel and a blank of the respective channel. Kinetic parameters were determined from the association phase (1 to 120 s) and dissociation phase (120 to 300 s) of the SPR sensorgrams. The y-axis displays the SPR response in response units (RU). Chemokine accumulation on **J** heparin, **K** HS and **L** CS-A. The maximum signal (Rmax; RU) obtained during injection of CXCL4, CXCL10_(1–77)_ and CXCL10_(1–73)_ was plotted in function of the chemokine concentration. Data were plotted as the mean (± SEM) of 3–4 independent experiments

Given the potential involvement of the positively charged C-terminal amino-acids Lys^74^ and Arg^75^ of CXCL10 in binding to GAGs, we investigated binding of CXCL10_(1–73)_ and intact CXCL10_(1–77)_ to heparin, heparan sulfate (HS) and chondroitin sulfate (CS)-A using surface plasmon resonance (SPR) (Fig. 2A-L). CXCL4 was included as a positive control for CS-A binding [62]. Varying concentrations of CXCL10_(1–77)_, CXCL10_(1–73)_ and CXCL4 were sent over a neutravidin-coated CM4 chip on which distinct GAGs were immobilized in individual flow channels (Fig. 2A). To characterize the nature of the interaction, kinetic parameters were determined from the association (1 to 120 s) and dissociation (120 to 300 s) phases of the SPR sensorgrams (Table 1). Given the acknowledged non-ideal behavior of chemokines for SPR analysis [63], binding kinetics were analyzed and fitted through the 1:1 binding model with mass transfer correction [63, 64] to calculate “apparent K_D_ values” (Fig. S2). Compared to CXCL10_(1–77)_, CXCL10_(1–73)_ showed 32.4-fold reduced affinity for heparin (Fig. 2B, C, Table 1) and 3.7-fold decreased affinity for HS (Fig. 2E, F, Table 1). Furthermore, CXCL10_(1–73)_ bound weakly and 15.3-fold less efficient to CS-A compared to CXCL10_(1–77)_ (Fig. 2H, I, Table 1). As expected, CXCL4 exhibited higher affinity for HS and CS-A compared to CXCL10_(1–77)_ (Fig. 2D, E and G, H, Table 1). Thus, CXCL10_(1–73)_ displayed diminished affinity for HS, CS-A and heparin compared to CXCL10_(1–77)_. In addition, we evaluated maximal surface accumulation on GAGs of these CXCL10 proteoforms (Fig. 2J-L), thereby plotting the maximal signal produced by these chemokines in function of the chemokine concentration [63]. CXCL4 reached the highest level of accumulation on HS and CS-A (Fig. 2K, L). CXCL10_(1–73)_ required higher concentrations to reach comparable maximal RU levels compared to CXCL10_(1–77)_, which is consistent with the lower affinity interactions with GAGs (Fig. 2J-L).

**Table 1:** Kinetic parameters of the interaction between human CXCL10 proteoforms and GAGs

	**Heparan sulfate**			**Heparin**			**Chondroitin sulfate A**		
Chemokine	k_on_ (1/M.s)	k_off_ (1/s)	Apparent K_D_ (nM)	k_on_ (1/M.s)	k_off_ (1/s)	Apparent K_D_ (nM)	k_on_ (1/M.s)	k_off_ (1/s)	Apparent K_D_ (nM)
CXCL4	(8.58 ± 0.54) E+ 05	(3.21 ± 0.14) E-03	3.75 ± 0.10	N.D.	N.D.	N.D.	(4.36 ± 0.60) E+ 05	(6.71 ± 0.77) E-03	15.91 ± 2.61
CXCL10_(1–77)_	(5.28 ± 0.84) E+ 05	(3.41 ± 0.43) E-03	6.73 ± 0.65	(9.76 ± 0.41) E+ 05	(1.00 ± 0.04) E-03	1.03 ± 0.01	(1.69 ± 0.09) E+ 05	(5.57 ± 0.34) E-03	33.40 ± 2.86
CXCL10_(1–73)_	(4.86 ± 0.96) E+ 04	(1.26 ± 0.27) E-03	25.23 ± 1.16	(4.26 ± 0.32) E+ 04	(1.39 ± 0.04) E-03	33.42 ± 2.65	(3.15 ± 1.08) E+ 03	(1.06 ± 0.12) E-03	512.40 ± 191.31

Values represent mean ± SEM of 3 to 5 independent experiments. Kinetic parameters were determined from the association phase (1 to 120 s) and dissociation phase (120 to 300 s) of the SPR sensorgrams. The apparent K_D_ was calculated from the ratio of k_off_ over k_on_ (nM) determined by the 1:1 binding model with mass transfer correction. k_on_ association rate constant (M^−1^ s^−1^); k_off_ dissociation rate constant (s^−1^); K_D_ dissociation equilibrium (affinity) constant

*N.D.* not determined

### CXCL10_(1–73)_ is a less potent inducer of second messenger signaling downstream of CXCR3A and chemotaxis of CXCR3^+^ T lymphocytes compared to intact CXCL10_(1–77)_

**Fig. 3:**
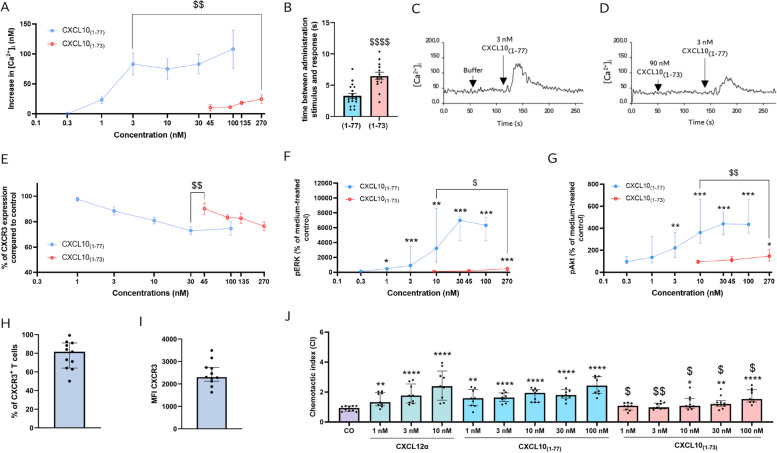
C-terminally truncated CXCL10_(1–73)_ has less potent signaling and chemotactic capacity in CXCR3A-transfected CHO cells and T lymphocytes. **A** CXCL10_(1–77)_ and CXCL10_(1–73)_ were evaluated for their ability to induce an increase of the intracellular calcium concentration ([Ca^2+^]_i_) in CXCR3A-transfected CHO cells. Results are displayed as mean (± SEM) increase of [Ca^2+^]_i_ of 4 independent experiments. Responses induced by 3 nM CXCL10_(1–77)_ and 270 nM CXCL10_(1–73)_ were compared using an unpaired t-test ($$ *p* ≤ 0.01). **B** Time between administration of the stimulus and response in sec (s). Results are displayed as mean (± SEM) of 3 independent experiments. Statistically significant differences between CXCL10_(1–77)_ and CXCL10_(1–73)_ were determined by an unpaired t-test ($$$$ *p* ≤ 0.0001). **C** and  **D** Representative curves show desensitization of CXCR3A-mediated [Ca^2+^]_i_ mobilization upon stimulation with 3 nM CXCL10_(1–77)_ following treatment with CXCL10_(1–73)_ or buffer as first stimulus. **E** Relative surface expression of CXCR3 on primary T lymphocytes activated with PHA and IL-2 (compared to medium-treated control cells) following stimulation with CXCL10_(1–77)_ and CXCL10_(1–73)_. Results are shown as median (± SEM) of 3 independent experiments with 9 different cell preparations in total. Responses induced by 30 nM CXCL10_(1–77)_ and 45 nM CXCL10_(1–73)_ were compared using an unpaired t-test ($$ *p* ≤ 0.01). **F**, **G** Levels of ERK1/2 and Akt phosphorylation in CXCR3A-transfected CHO cells stimulated with CXCL10 proteoforms. Results are shown as median (± IQR) of 4 to 8 independent experiments. Statistically significant ERK1/2 and Akt phosphorylation induced by CXCL10_(1–77)_ and CXCL10_(1–73)_ compared to medium-treated cells were determined by Mann–Whitney U test (* *p* ≤ 0.05, **, *p* ≤ 0.01, *** *p* ≤ 0.001). Comparison of the ERK1/2 and Akt phosphorylation induced by 10 nM CXCL10_(1–77)_ and 270 nM CXCL10_(1–73)_ was also performed through a Mann–Whitney U test ($ *p* ≤ 0.05, $$, *p* ≤ 0.01). **H** CXCR3 expression on PHA- and IL-2-stimulated T lymphocytes (gated as CD3^+^ CD56^−^ CD19^−^ cells) was evaluated through flow cytometry with proportions of CXCR3^+^ T lymphocytes and **I** MFI of CXCR3. **J** Chemotactic index (CI) showing migration of PHA- and IL-2-stimulated T lymphocytes after treatment with medium (HBSS + 0.1% BSA) as control condition (CO) or serial dilution of CXCL10_(1–77)_ (100 nM to 1 nM) or CXCL10_(1–73)_ (100 nM to 1 nM). Results are shown as median (± IQR) of 4 independent experiments with 10 different cell preparations in total. Statistically significant CI compared to medium-treated cells (* *p* ≤ 0.05, **, *p* ≤ 0.01, *** *p* ≤ 0.001, **** *p* ≤ 0.0001) or between CXCL10 proteoforms ($ *p* ≤ 0.05, $$ *p* ≤ 0.01) were determined by Mann–Whitney U test

In calcium assays, CXCR3A-transfected CHO cells were used to determine whether CXCL10_(1–73)_ had a similar potency as CXCL10_(1–77)_ to induce mobilization of intracellular calcium. Solely high concentrations of CXCL10_(1–73)_ (i.e., 135 nM and 270 nM) were able to induce an increase in intracellular calcium concentrations, thereby reaching comparable levels as upon stimulation with 1 nM CXCL10_(1–77)_ (Fig. 3A). At 3 nM of CXCL10_(1–77)_, calcium mobilization was even significantly higher compared to 270 nM of CXCL10_(1–73)_. We also observed that the time between administration of the stimulus and the initiation of the calcium increase was prolonged for CXCL10_(1–73)_ independent of the administrated dose (Fig. 3B). The markedly limited capacity of CXCL10_(1–73)_ compared to CXCL10_(1–77)_ to mobilize intracellular calcium sparks the notion of potential CXCR3 desensitization by this C-terminally shortened CXCL10 proteoform at inactive concentrations. Indeed, when 100 s prior to a stimulus of 3 nM CXCL10_(1–77)_, 45 nM or 90 nM of inactive CXCL10_(1–73)_ was added to the cells, the increase of intracellular calcium upon stimulation with CXCL10_(1–77)_ was reduced by 69.9% and 72.2%, respectively (Fig. 3C, D). CXCR3 desensitization may be due to partial agonism or receptor internalization. Therefore, we evaluated the effects of both CXCL10 proteoforms on CXCR3 internalization on primary T lymphocytes derived from PBMCs of individual donors and stimulated with phytohemagglutinin (PHA) and IL-2. CXCL10_(1–77)_ induced CXCR3 internalization more potently compared to CXCL10_(1–73)_, as the relative surface expression of CXCR3A was significantly further reduced upon incubation with 30 nM CXCL10_(1–77)_ compared to 45 nM CXCL10_(1–73)_ (Fig. 3E). In addition, we found that limited internalization of CXCR3 was induced by concentrations of CXCL10_(1–73)_ that were able to desensitize CXCR3A, i.e. 45 nM (mean MFI of CXCR3 =  90.1%) and 90 nM (mean MFI of CXCR3 = 83.3%). Hence, receptor internalization and partial agonism are both likely to contribute to CXCR3A desensitization mediated by CXCL10_(1–73)_.

Second, the ability of CXCL10_(1–73)_ to induce phosphorylation of ERK1/2 and protein kinase B/Akt in CXCR3A-transfected CHO cells was tested. Similar to the calcium experiments, high concentrations of CXCL10_(1–73)_ (270 nM) only weakly induced phosphorylation of ERK1/2 and Akt, thereby inciting comparable median levels of phosphorylated ERK1/2 (pERK1/2) and phosphorylated Akt (pAkt) as induced by 1 nM of CXCL10_(1–77)_ (Fig. 3F, G). Furthermore, significantly increased pAkt and pERK levels were observed upon treatment with 10 nM of CXCL10_(1–77)_ compared to 270 nM of CXCL10_(1–73)_. Hence, CXCL10_(1–73)_ exhibited reduced potency compared to CXCL10_(1–77)_ to induce intracellular calcium mobilization, internalization of CXCR3A and phosphorylation of ERK1/2 and Akt.

Given that the C-terminal truncation of CXCL10 significantly attenuated CXCR3 signaling, we further investigated whether CXCL10_(1–73)_ also had reduced T lymphocyte chemotactic capacities. To ascertain adequate responsiveness of T lymphocytes, CXCL12α was included as positive control. T lymphocytes stimulated with PHA and IL-2 are known to express CXCR4 and pronouncedly migrate after exposure to CXCL12α [65]. Most T lymphocytes (median CXCR3 expression = 81.6%, median MFI of CXCR3 = 2303) were positive for CXCR3 (Fig. 3H, I; gating shown in Suppl. Fig. S3). Given that in vitro T cell chemotaxis induced by CXCL10 has been shown to occur in the absence of coating [47] and to exclude that distinct binding affinities of the CXCL10 proteoforms to extracellular matrix proteins underlie the difference in T lymphocyte migration, we evaluated migration through uncoated -membranes. We confirmed that chemotaxis was significantly and dose-dependently increased upon stimulation with CXCL12α (Fig. 3J). Starting from 1 nM, CXCL10_(1–77)_ induced a significant and dose-dependent migration of CXCR3^+^ T lymphocytes compared to cells exposed to buffer. The migratory response of T lymphocytes towards CXCL10_(1–73)_ was significantly increased compared to buffer from 10 nM CXCL10_(1–73)_ onwards. Chemotaxis of T cells was significantly diminished for CXCL10_(1–73)_ compared to CXCL10_(1–77)_ at all tested concentrations.

CXCL10 requires presentation on GAGs to mediate transendothelial migration of primary human CD4^+^ T lymphocytes under conditions of physiological shear stress [66]. Hence, we also evaluated migration through membranes coated with different extracellular matrix proteins (i.e., bovine fibronectin [FN], human FN and human type I collagen). Upon coating with these proteins, CXCL10_(1–73)_ also induced significantly less T lymphocyte migration compared to CXCL10_(1–77)_ at 10 nM, 30 nM and/or 100 nM (Fig. S4A-C) with no significant differences between human FN and human type I collagen. Thus, in line with the observation of the signaling assays, C-terminal processing of CXCL10 also significantly attenuates its chemotactic properties on primary human CXCR3^+^ T lymphocytes.

### CXCL10_(1–73)_ is equally potent in exerting antiangiogenic actions compared to intact CXCL10_(1–77)_

Since the CXCL10-derived peptide CXCL10_(56–77)_ was reported to be equally potent in mediating angiostatic effects as intact CXCL10_(1–77)_ [43], one could presume that CXCL10_(1–73)_ has attenuated capacity to exert anti-angiogenic actions. For this reason, we examined the activities of CXCL10_(1–73)_ on human microvascular endothelial cells (HMVEC) in migration, proliferation, wound closure, signal transduction and sprouting assays.

**Fig. 4:**
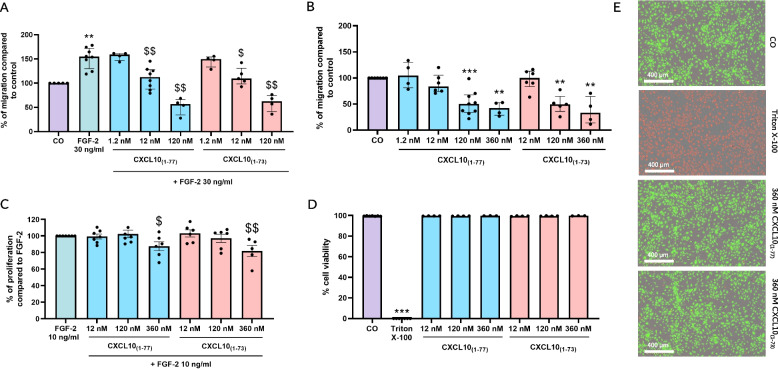
Equipotent inhibition of spontaneous and FGF-2-induced HMVEC migration by intact CXCL10_(1–77)_ or C-terminally truncated CXCL10_(1–73)_ without exerting cellular toxicity. HMVEC chemotaxis was measured towards **A** 30 ng/ml FGF-2 and **B** EBM-2 $$+$$ 0.4% FCS treated cells (CO) in the presence or absence of CXCL10_(1–77)_ or CXCL10_(1–73)_. The data are displayed as median (± IQR) of 4 to 7 independent experiments. Statistically significant differences in migration compared to cells treated with control medium or FGF-2 were determined by a Mann–Whitney U test (** *p* ≤ 0.01, *** *p* ≤ 0.001 for comparison to control, $$ *p* ≤ 0.01 for comparison to FGF-2). **C** FGF-2-induced proliferation of HMVEC was examined in the presence or absence of CXCL10_(1–77)_ or CXCL10_(1–73)_. The data are displayed as mean (± SEM) of 5 to 7 independent experiments. Statistically significant differences in proliferation compared to cells treated with 10 ng/ml FGF-2 were determined by an unpaired t-test ($ *p* ≤ 0.05; $$ *p* ≤ 0.01). **D** Cellular toxicity was assessed after 30 h of stimulation with CXCL10_(1–77)_ or CXCL10_(1–73)_. The median (± IQR) of 3 to 4 independent experiments is shown. Statistically significant differences in cell viability compared to cells treated with control were determined by a Mann–Whitney U test (*** *p* ≤ 0.001). **E** Representative images are displayed of HMVEC treated with control medium (CO; MCDB131 + 0.4% [*v/v*] FCS), 2% (*v/v*) Triton X-100 to induce cell death, and 360 nM of CXCL10_(1–77)_ or CXCL10_(1–73)_. Scale bar = 400 µm

First, chemotactic migration of endothelial cells in the presence of CXCL10 proteoforms was evaluated. Migration of HMVEC was monitored and analyzed at 12 h to exclude potential anti-proliferative effects of CXCL10. Stimulation with FGF-2 caused a significant increase of migration of endothelial cells (Fig. 4A). CXCL10_(1–73)_ was equally potent as intact CXCL10_(1–77)_ in inhibiting FGF-2-induced chemotaxis of HMVEC. Starting from 12 nM, both CXCL10 isoforms suppressed FGF-2-mediated migration of endothelial cells in a dose-dependent manner. Accordingly, spontaneous HMVEC chemotaxis was dose-dependently inhibited with similar efficiency for both CXCL10 proteoforms from a concentration of 120 nM onwards (Fig. 4B). Although spontaneous migration was slightly decreased by 12 nM CXCL10_(1–77)_, both CXCL10 proteoforms were not able to significantly counteract spontaneous chemotaxis of endothelial cells at a dose of 12 nM in contrast to the FGF-2-induced migration.

Second, we examined the effects of both CXCL10 proteoforms on proliferation of HMVEC. Both CXCL10_(1–73)_ and CXCL10_(1–77)_ equally inhibited FGF-2-induced proliferation at 360 nM (Fig. 4C). To ascertain that the inhibitory effects of CXCL10 proteoforms were not due to cellular toxicity, their effects on endothelial cell survival were investigated. At the highest evaluated concentration (360 nM), CXCL10_(1–73)_ and CXCL10_(1–77)_ did not affect the viability of HMVEC after incubation for 30 h (Fig. 4D, E).

**Fig. 5:**
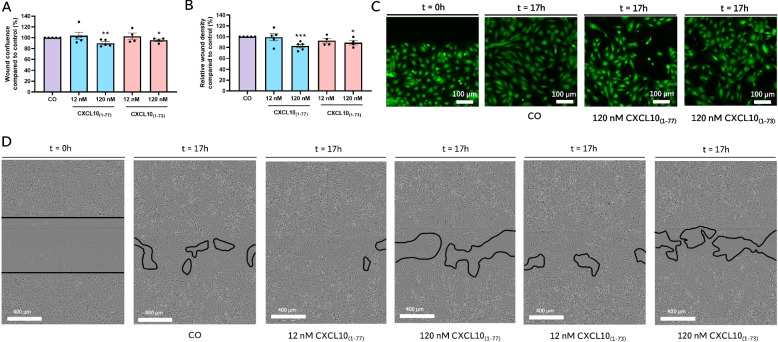
Equivalent inhibition of spontaneous HMVEC migration and invasion by intact CXCL10_(1–77)_ or C-terminally truncated CXCL10_(1–73)_. After creating a scratch wound, spontaneous HMVEC migration and invasion was monitored for 17 h in EBM-2 $+$ 1% FCS (CO) in the presence or absence of CXCL10_(1–77)_ or CXCL10_(1–73)_ using the IncuCyte S3 Live-Cell Analysis System. Percentages of **A** relative wound density and **B** wound confluence compared to medium-treated cells were represented in bar plots. The data are displayed as mean (± SEM) of 4 to 7 independent experiments. Unpaired t-test was used to compare differences in relative wound density and wound confluence compared to EBM-2 $+$ 1% FCS treated cells (CO) (* *p* ≤ 0.05, ** *p* ≤ 0.01, *** *p* ≤ 0.001). **C** Representative images of the wound borders using immunofluorescence microscopy after calcein staining of HMVEC stimulated with EBM-2 $+$ 1% FCS (CO), CXCL10_(1–77)_ or CXCL10_(1–73)_ at 120 nM. Scale bar = 100 µm. **D** Representative images of the full wound area using IncuCyte time-lapsed microscopy pictures of HMVEC stimulated with EBM-2 $+$ 1% FCS (CO), CXCL10_(1–77)_ or CXCL10_(1–73)_ at 12 nM and 120 nM. Scale bar = 400 µm

Third, we assessed the influence of CXCL10_(1–73)_ and CXCL10_(1–77)_ on the ability of endothelial cells to spontaneously invade and migrate into a scratch wound in a confluent monolayer. CXCL10_(1–73)_ and CXCL10_(1–77)_ suppressed spontaneous wound closure at a dose of 120 nM, marked by significantly attenuated relative wound density and wound confluence (Fig. 5A, B). Both CXCL10 proteoforms were not able to suppress spontaneous migration and invasion of endothelial cells at a dose of 12 nM. Differences in wound confluence were also observed after imaging the wound area (Fig. 5C, D; Fig. S5). In addition, CXCL10 proteoforms suppressed FGF-2-induced wound closure at 360 nM, but not at 120 nM (Fig. S6). Although spontaneous and FGF-2-induced wound closure was equivalently suppressed by both CXCL10 proteoforms, the effect size was limited. The apparent difference of the effects of CXCL10 proteoforms on FGF-2-induced migration at 12 nM observed through the xCELLigence migration assay may be explained by the limited resolution of the wound closure assay as opposed to the migration assay (i.e. accurate measurement of electrical impedance).

**Fig. 6:**
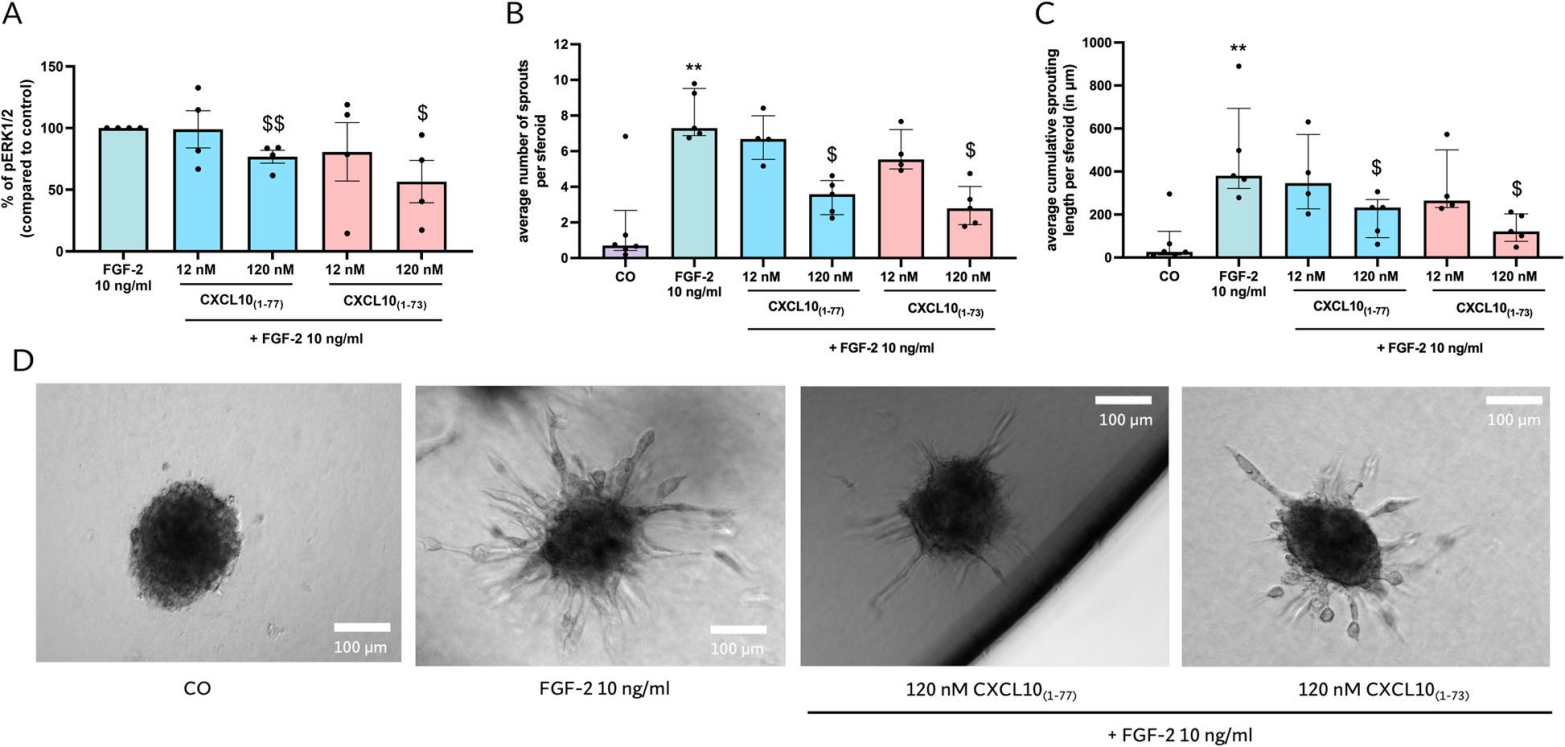
Equipotent inhibition of FGF-2-induced pERK1/2 signaling and spheroid sprouting by intact CXCL10_(1–77)_ or C-terminally truncated CXCL10_(1–73)_. **A** Phosphorylation of ERK1/2 was evaluated after 5 min stimulation of HMVEC with FGF-2 (10 ng/ml) in the absence or presence of CXCL10_(1–77)_ or CXCL10_(1–73)_. The data are displayed as mean (± SEM) of 4 independent experiments. Unpaired t-test was performed ($ *p* ≤ 0.05, $$ *p* ≤ 0.01 for comparison to FGF-2). Sprouting of collagen-embedded HMVEC spheroids was assessed upon stimulation with control medium EBM-2 + 3% FCS (CO), 10 ng/ml FGF-2 alone or with CXCL10_(1–77)_ or CXCL10_(1–73)_ at the indicated doses after 16 h incubation at 37 °C and 5% CO_2_. **B** Average number of sprouts per spheroid and **C** average cumulative sprouting length per spheroid (in µm) were determined with Fiji Software. The data are displayed as median (± IQR) of 4 to 5 independent experiments. Mann–Whitney U test was performed (** *p* ≤ 0.01 for comparison to control, $ *p* ≤ 0.05 for comparison to FGF-2). **D** Representative images of spheroids that were untreated (incubated with control medium EBM-2 + 3% FCS; CO), incubated with 10 ng/ml FGF-2 in the presence or absence of 120 nM of CXCL10_(1–77)_ or CXCL10_(1–73)_ are displayed. Scale bar = 100 µm

Fourth, we examined the ability of CXCL10 proteoforms to blunt the FGF-2-induced ERK signal transduction pathway. CXCL10_(1–73)_ and CXCL10_(1–77)_ significantly diminished FGF-2-induced ERK phosphorylation at 120 nM with no significant differences between the two proteoforms (Fig. 6A). In addition, we evaluated the effects of both CXCL10 proteoforms in the in vitro spheroid sprouting assay, in vivo which enables to assess angiogenesis in a 3-dimensional environment [67, 68]. Pronounced sprouting of collagen-embedded HMVEC spheroids was observed after treatment with 10 ng/ml FGF-2 for 16 h (Fig. 6B-D). CXCL10_(1–77)_ and CXCL10_(1–73)_ at concentrations of 120 nM efficiently diminished the FGF-2-induced number of sprouts that outgrew and reduced the cumulative sprout length of spheroids (Fig. 6B-D). In summary, these in vitro findings demonstrate that CXCL10_(1–77)_ and CXCL10_(1–73)_ have a comparable potency to suppress spontaneous and growth factor-induced angiogenic actions including endothelial cell migration, proliferation, wound closure, signal transduction and spheroid sprouting.

### CXCL10_(1–73)_ induces less in vivo migration of CXCR3^+^ lymphocytes compared to intact CXCL10_(1–77)_

**Fig. 7:**
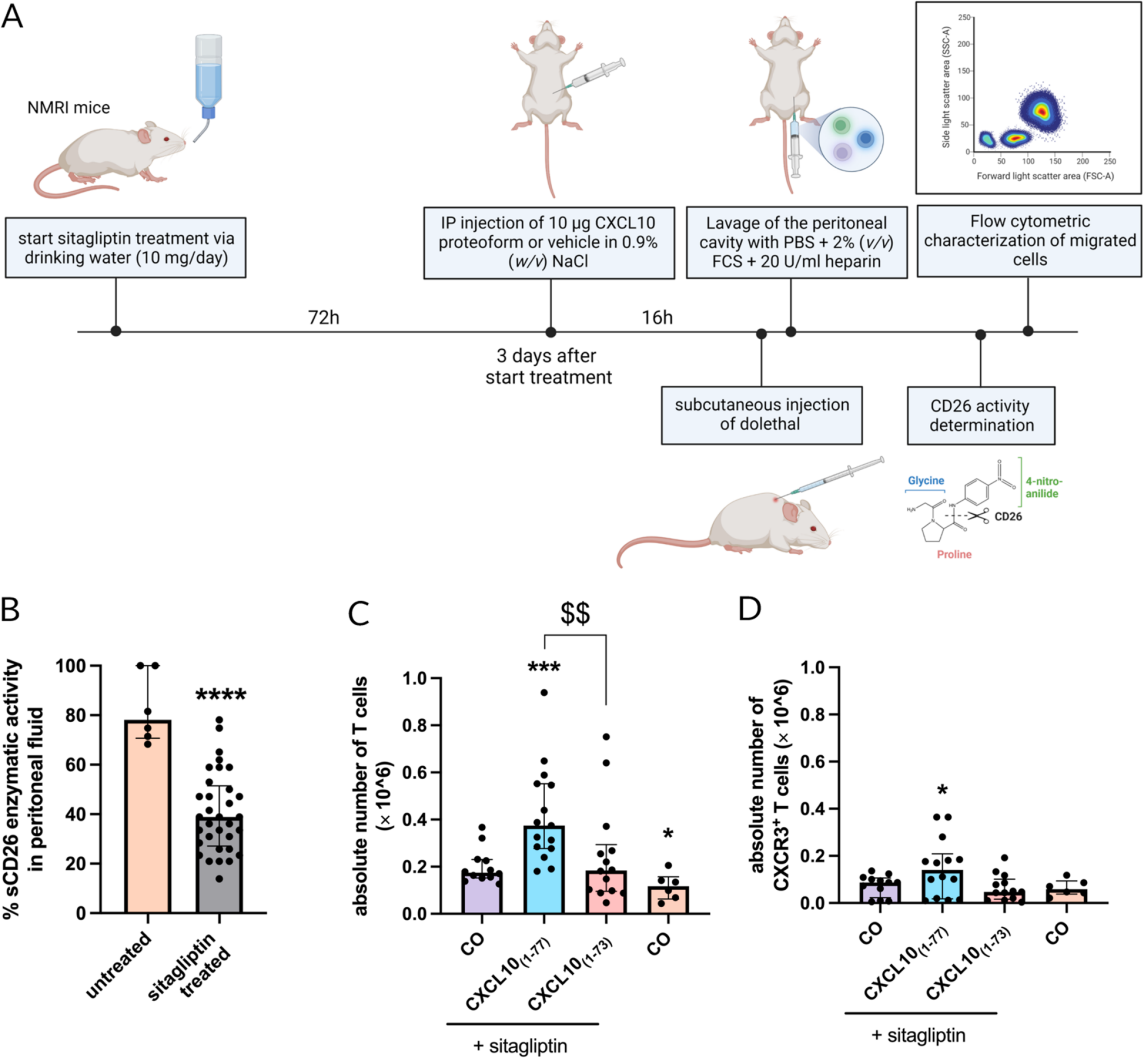
C-terminally truncated CXCL10_(1–73)_ evokes less in vivo migration of CXCR3^+^ T lymphocytes upon IP injection compared to intact CXCL10_(1–77)_. **A** Schematic representation of the experimental set-up. Female NMRI mice received sitagliptin via drinking water for 72 h (10 mg/day) and were intraperitoneally injected with vehicle (CO), 10 µg CXCL10_(1–77)_ or 10 µg CXCL10_(1–73)_ dissolved in 0.9% (*w/v*) NaCl 16 h prior to lavage of the peritoneal cavity. Migrated cells were analyzed through flow cytometry. Inhibition of soluble CD26 (sCD26) activity in the peritoneal lavage fluids was verified in a CD26 activity assay. **B** sCD26 enzymatic activity (%) in peritoneal lavage fluids of mice treated with sitagliptin and untreated mice. **C** Absolute numbers of T cells (gated as CD3^+^ NK1.1^−^) and **D** of activated CXCR3^+^ T cells (gated as CD3^+^ NK1.1^−^ CXCR3^+^) were determined. Each symbol represents an individual mouse (n ≥ 6 per group). Four independent experiments were performed. Horizontal lines and error bars mark the median number of cells with interquartile range. Statistical analysis was performed using a Mann–Whitney U test (* *p* ≤ 0.05, *** *p* ≤ 0.001 for comparison to sitagliptin-treated control mice, $$ *p* ≤ 0.01 for comparison of CXCL10_(1–73)_ to CXCL10_(1–77)_)

**Fig. 8:**
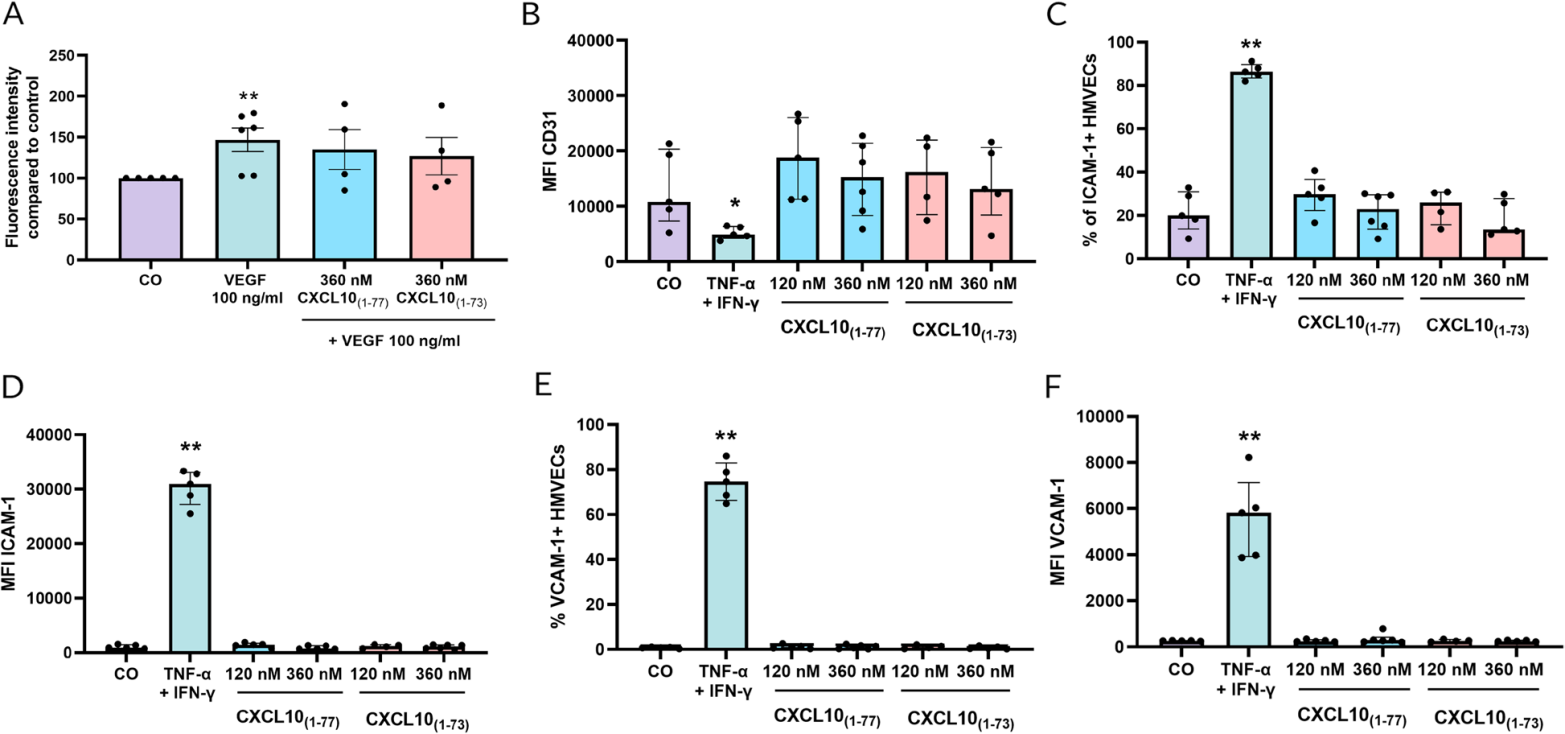
Permeability and lymphocyte adhesion molecule expression of HMVEC is not increased by C-terminally truncated CXCL10_(1–73)_ or intact CXCL10_(1–77)_. **A** Endothelial monolayer permeability was assessed after stimulation with control medium (CO), or stimulated with VEGF (100 ng/ml) alone or VEGF (100 ng/ml) combined with 360 nM CXCL10_(1–77)_ or CXCL10_(1–73)_. Data are displayed as median (± IQR) of 4 to 6 independent experiments. Mann–Whitney U test was performed (* *p* ≤ 0.05, ** *p* ≤ 0.01 for comparison to control). Expression and/or MFI of **B** PECAM-1/CD31, **C**, **D** ICAM-1/CD54, and **E**, **F** VCAM-1/CD106 on HMVEC (gated as CD31^+^ cells) was evaluated through flow cytometry upon stimulation for 48 h at 37 °C and 5% CO_2_ with control medium (CO), 100 ng/ml TNF-α and 100 ng/ml IFN-γ, or CXCL10_(1–77)_ or CXCL10_(1–73)_ at the indicated doses. The data are displayed as median (± IQR) of 4 to 6 independent experiments. Mann–Whitney U test was performed (* *p* ≤ 0.05, ** *p* ≤ 0.01 for comparison to control)

Chemokine injection into the peritoneal cavity of NMRI mice followed by peritoneal lavage was used as an experimental model to examine the in vivo ability of CXCL10_(1–73)_ to attract leukocytes. Mice received the competitive CD26 inhibitor sitagliptin via the drinking water for 72 h prior to chemokine injection (Fig. 7A) to avoid CD26-mediated cleavage [35, 69]. Sitagliptin thereby preserves the integrity of the N-terminus of CXCL10 and its ability to induce lymphocyte attraction after intraperitoneal (IP) injection in mice [35]. This allows clear evaluation of the effects of the C-terminal truncation. Mice had an estimated average intake of 10 mg/day of sitagliptin via the drinking water (Fig. S7A). To accurately determine the residual CD26 activity in the peritoneal lavage fluids of sitagliptin-treated mice, a calibration curve was established of the percentage of CD26 activity in function of the sitagliptin concentration (Fig. S7B). The percentage of soluble CD26 enzymatic activity in the peritoneal lavage fluid of sitagliptin-treated mice was significantly diminished compared to lavage fluids of untreated mice (Fig. 7B), confirming CD26 inhibition at the site of chemokine injection. Furthermore, immunophenotyping of peritoneal leukocytes was performed via flow cytometry. Injection of 10 $\mu$g of CXCL10_(1–77)_, but not CXCL10_(1–73)_, significantly augmented the recruitment of T cells towards the peritoneal cavity compared to vehicle-treated mice (Fig. 7C). In addition, absolute cell numbers of CXCR3^+^ T cells were increased —although to a limited extent— in the peritoneal lavage fluids of CXCL10_(1–77)_-treated mice (Fig. 7D). Increased proportions or trends towards increased proportions of T cells, CD4^+^ T cells, NKT cells, and B cells, and their activated CXCR3^+^ subsets were also found for CXCL10_(1–77)_-treated mice, but not for littermates receiving CXCL10_(1–73)_ (Fig. S8A-H). However, lymphocyte trafficking in vivo can also be affected by changes in vascular permeability and lymphocyte adhesion molecules. Therefore, we assessed whether CXCL10 proteoforms influenced vascular permeability of confluent monolayers of HMVEC (Fig. 8A). Confluence of the monolayers on the transwell inserts was confirmed through confocal microscopy (Fig. S9A). Both CXCL10 proteoforms at 360 nM did not affect VEGF-induced vascular permeability (Fig. 8A). We also examined whether CXCL10 proteoforms altered the presence of lymphocyte adhesion molecules, tight junctions, or adherence junctions on HMVEC. PECAM-1/CD31 was significantly decreased by combined treatment with 100 ng/ml TNF-α and 100 ng/ml IFN-γ (Fig. 8B) as previously reported [70]. CXCL10_(1–77)_ and CXCL10_(1–73)_ did not affect the expression of PECAM-1. Combined treatment with 100 ng/ml TNF-α and 100 ng/ml IFN-γ significantly augmented the expression of lymphocyte adhesion molecules including intracellular adhesion molecule 1 (ICAM-1) and vascular cell adhesion molecule 1 (VCAM-1) (Fig. 8C-F). Again, both CXCL10 proteoforms did not affect expression of ICAM-1 (Fig. 8C, D) and VCAM-1 (Fig. 8E, F) upon 48 h incubation. Furthermore, the two CXCL10 proteoforms did not affect the expression of adherence junction vascular endothelial [VE]-cadherin (Fig. S9B, C) nor tight junction zona occludens 1 (ZO-1) (Fig. S9D, E). Hence, these findings provide further evidence that CXCL10_(1–73)_ is less potent at inducing T lymphocyte chemotaxis in vivo compared to CXCL10_(1–77)_, presumably by a direct effect on CXCR3^+^ T lymphocytes.

## Discussion

In the present study, we characterized the effects of a synthetic CXCL10 proteoform corresponding to natural C-terminally truncated CXCL10_(1–73)_ that was previously identified in human cell culture supernatant [27, 29, 30]. The detection of natural CXCL10_(1–73)_ in synovial fluids of RA patients urged us to develop a strategy for Fmoc-based SPPS of CXCL10_(1–73)_ to ensure the availability of sufficient amounts of the pure proteoform for its biological characterization. CXCL10_(1–77)_ was previously generated through SPPS based on *tertiary* butyloxycarbonyl (*t*Boc) chemistry [61, 71]. However, major drawbacks of Boc chemistry include the use of corrosive trifluoroacetic acid (TFA) in the synthesizer for removal of N-terminal Boc protection groups and the hazardous hydrofluoric acid (HF) for peptide cleavage from the solid phase support [72]. These obstacles were surmounted via Fmoc chemistry [72], whereby Fmoc protection groups are removed under moderate basic conditions and cleavage of peptides from the resin is performed via TFA. However, the moderate hydrophobic nature of CXCL10_(1–73)_ hampered correct Fmoc-based SPPS, resulting in a poor yield with highly abundant contaminants, consisting of incompletely synthesized peptides. Indeed, proteins comprising of a high number of amino acids possessing hydrophobic side chains are termed “difficult peptides”, given their profound challenges and complications in terms of their synthesis and purification [73, 74]. These proteins tend to form inter- and intra-molecular β-sheet interactions, resulting in on-resin aggregation during peptide synthesis and consequently synthesis failure. To overcome this major obstacle, the concomitant use of pseudoproline dipeptides and a hydrophilic polyethylene glycol (PEG) resin was explored. This strategy was previously shown to substantially increase the synthesis yield of the human chemokine RANTES/CCL5_(1–68)_ [75]. In addition, we used a high quality coupling system, i.e., HCTU and NMM [58–60], as described in a recently established methodology for Fmoc-based SPPS of mCXCL10_(1–77)_ [60]. The latter authors also utilized a pseudoproline dipeptide at Ala^43^-Thr^44^, in addition to other modifications compared to the ones described in this study, as challenging protein regions differed between human CXCL10_(1–73)_ and mCXCL10_(1–77)_. For successful Fmoc synthesis of human CXCL10_(1–73)_, incorporation of an Fmoc-Ile-Pro dimer at position Ile_30_-Pro_31_ and three additional pseudoprolines (at positions Arg^5^-Thr^6^, Ile^12^-Ser^13^, and Val^68^-Ser^69^) was essential. The established SPPS approach for human CXCL10_(1–73)_ can be easily extrapolated towards other CXCL10 proteoforms. N-terminal truncations and an intact C-terminus can be incorporated through earlier termination of the synthesis and usage of a 2-chlorotrityl resin to prevent diketopiperazine formation associated with a C-terminal Pro [60], respectively.

A former research effort aiming to study native C-terminally truncated CXCL10_(1–73)_ was made by Hensbergen et al*.* [29]. They opted for the use of recombinant CXCL10_(1–73)_ with an additional N-terminal methionine (Met-CXCL10_(1–73)_) [29], which is an artefact due to the expression of the chemokine in bacteria. Met-CXCL10_(1–73)_ was generated via furin- and carboxypeptidase B-mediated C-terminal cleavage of recombinant Met-CXCL10_(1–77)_ [29]. Met-CXCL10_(1–73)_ retained equal potency to Met-CXCL10_(1–77)_ to induce chemotaxis of primary human T cells stimulated with PHA and IL-2, Gα and intracellular calcium signaling in CXCR3-transfected CHO cells, and inverse agonism on the human herpes virus 8 (HHV-8)-associated ORF74 receptor [29]. In contrast, we observed significantly attenuated intracellular calcium signaling, ERK and PKB/Akt phosphorylation evoked by synthetic CXCL10_(1–73)_, indicating that second messenger signaling downstream of CXCR3A through Gαq and Gβγ is severely affected by the C-terminal truncation. These contrasting findings may be explained by the fact that CXCL10-mediated calcium mobilization and chemotaxis is known to be strongly impaired by the presence of an additional N-terminal Met in the primary sequence of CXCL10 [34]. Hence, Met-CXCL10_(1–73)_ may be an inadequate substitute for natural human CXCL10_(1–73)_. In addition, our findings accorded with data of Antonia et al*.* demonstrating that a CXCL10 proteoform lacking the α-helical and coiled C-terminal residues displayed significantly impaired chemotaxis of CXCR3^+^ Jurkat T cells in vitro [44].

**Fig. 9:**
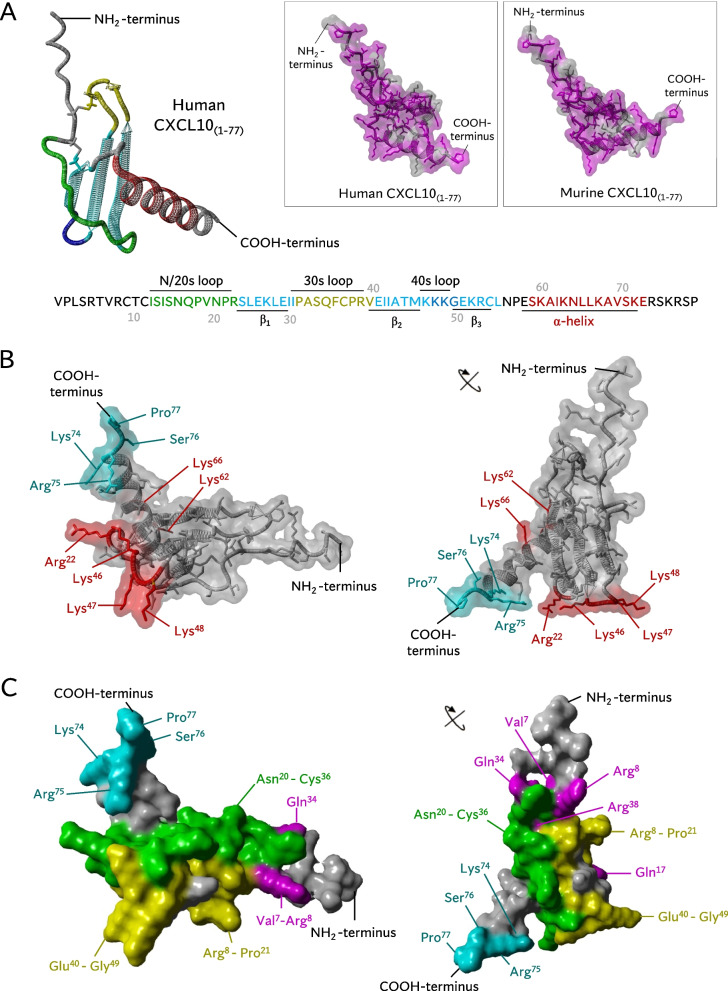
Structure models of human CXCL10_(1–77)_. **A** Structure models of human CXCL10_(1–77)_ of AlfaFold DB (right; AF-P02778-F1 without signal sequence). This model is based on the crystal structure of CXCL10 in hexagonal (H) form [PDB accession code 1O80], crystal structure of CXCL10 in monoclinic (M) form [PDB accession code 1O7Y], crystal structure of CXCL10 in tetragonal (T) form [PDB accession code 1O7Z], and NMR spectroscopy-determined CXCL10 [PDB accession code 1LV9] [45, 46] whereby unobserved Ser^76^ and Pro^77^ were predicted through AlfaFold DB. Secondary structures of human CXCL10_(1–77)_ are displayed. CXCL10 has an N/20s loop (green), three antiparallel β-sheets (cyan), 30s loop (yellow), 40s loop (blue), and an α-helix (red). The inset shows human CXCL10_(1–77)_ (AF-P02778-F1; left) and murine CXCL10_(1–77)_ of AlfaFold DB (AF-Q3UK71-F1 without signal sequence; right). Conserved residues in mCXCL10_(1–77)_ and human CXCL10_(1–77)_ (magenta) and residues that are not conserved (grey) are displayed. **B** The structural model of human CXCL10_(1–77)_ of AlfaFold DB (AF-P02778-F1) is shown from two different perspectives with a transparent surface to visualize amino acid side chains. The four C-terminal residues that are shedded in CXCL10_(1–73)_ (cyan) are located in close proximity to predicted potential GAG-binding residues Arg^22^, Lys^46^, Lys^47^, Lys^48^, Lys^62^, and Lys^66^ (red) [46]. **C** The structural model of human CXCL10_(1–77)_ of AlfaFold DB (AF-P02778-F1) is displayed from two different perspectives with a non-transparent surface to visualize the receptor interaction surface. Potential CXCR3 binding residues of CXCL10 are indicated in different colors as previously shown by Swaminathan et al*.* [46]: residues of human CXCL10_(1–77)_ found to be perturbed in 2D ^15^N-^1^H HSQC NMR spectra by the addition of an N-terminal CXCR3 peptide CXCR3_(22–42)_ [45] (Val^7^, Arg^8^, Gln^17^, Val^19^, Gln^34^ and Arg^38^; magenta), residues bound by a CXCR3-blocking anti-CXCL10 monoclonal antibodies preventing chemotaxis and calcium mobilization [46] (Asn^20^-Cys^36^; green), and residues aligned to the CXCL8 binding region to CXCR1 (Arg^8^-Pro^21^ and Glu^40^-Gly^49^; yellow) [77]. The four C-terminal residues that are shedded in CXCL10_(1–73)_ (cyan) are positioned in vicinity of several potential CXCR3 binding residues

 Furthermore, a C-terminally mutated mCXCL10 containing K71E, R72Q, K74Q, and R75E exhibited reduced binding affinity for heparin and mouse CXCR3, diminished intracellular calcium mobilization, decreased chemotaxis of 300–19/mCXCR3 transfected cells, and an impaired ability to induce mCXCR3 internalization [47]. Mutant K71E/R72Q/K74Q/R75E mCXCL10 with an additional R22A mutation (C-tR22A) displayed an even more pronounced impairment of the aforementioned functional features [47]. Furthermore, this C-tR22A mCXCL10 mutant failed to execute hallmark properties of native mCXCL10_(1–77)_, including inhibition of proliferation of human umbilical vein endothelial cells (HUVEC) [48], cell surface binding of dengue virus to mouse hepatoma cells [49] and chemotactic migration of primary lung fibroblasts treated with bronchoalveolar lavage fluid (BALF) of bleomycin-treated mice [50]. These features were attributed to the attenuated GAG binding ability of C-tR22A mCXCL10 [47]. Mature secreted human CXCL10_(1–77)_ has 70.1% amino acid identity (54/77 amino acids) with mature secreted mCXCL10_(1–77)_ and their C-terminal α-helical and coiled residues Pro^56^-Pro^77^ show profound conservation (16/22 amino acids; 72.7%; Fig. 9A). Hence, our findings, showing that the loss of the two C-terminally located basic amino acids (Lys^74^ and Arg^75^) in human CXCL10_(1–73)_ diminishes the affinity for GAGs, is substantiated at multiple levels. Firstly, the evolutionary conserved positively charged Lys^74^ and Arg^75^ in human CXCL10 (although not being part of a paradigmatic GAG-binding chemokine motif BBXB or BBBXXBX [76]) may serve as GAG binding residues, as shown for mCXCL10 [47]. Secondly, Lys^74^ and Arg^75^ of the C-terminus are located in proximity to the Arg^22^ of the N/20s loop and 40s loop in the structure model of CXCL10_(1–77)_, similar to mCXCL10 [47]. Therefore, the C-terminal amino acids would be positioned adjacent to the platelet factor 4 (PF4/CXCL4)-based predicted GAG binding residues of CXCL10_(1–77)_ (Arg^22^, Lys^46^, Lys^47^, Lys^48^, Lys^62^, Lys^66^ [46]) as shown in Fig. 9B. As such, these C-terminal residues may constitute direct GAG binding or indirectly affect the other amino acids involved in GAG binding due to their vicinity. In addition to its reduced affinity for GAGs, we observed that CXCL10_(1–73)_ displayed attenuated CXCR3A signaling and diminished chemotactic potency for T lymphocytes. Booth et al. postulated that interaction of CXCL10 with CXCR3A would involve two demarcated hydrophobic clefts formed by the N loop and the 40s loop, in addition to the N-terminus and the 30s loop (Fig. 9A) [45]. More specifically, Val^7^, Arg^8^, Gln^17^, Gln^34^, Val^19^, and Arg^38^ would be responsible for CXCR3A binding based on the NMR structure (Fig. 9C) [45]. Additional partially overlapping CXCR3 binding regions were identified by others, including Asn^20^-Cys^36^ [46] and Arg^8^-Pro^21^ and Glu^40^-Gly^49^ [77] (Fig. 9C). Thus, Arg^22^ (20s loop), Lys^46^, Lys^47^ and Lys^48^ (40s loop) are likely involved in both GAG and receptor binding of CXCL10. Moreover, the initial high affinity binding of CXCL10 to CXCR3A is dependent on interaction with negatively charged sulfated Tyr^27^ and Tyr^29^ and N-glycosylated Asn^22^ and Asn^32^ in the N-terminal region of CXCR3A [78–80]. Hence, we hypothesize that the spatial vicinity of the positively charged C-terminal residues Lys^74^ and Arg^75^ and the N/20s-loop (in particular Arg^22^ implied in CXCR3A binding [46]) may form a Coulomb-assisted interaction surface to bind sulfo-Tyr^27/29^ and N-glycosylated Asn^22/32^ and thereby enable docking to CXCR3A. Presumably, the absence of C-terminal Lys^74^ and Arg^75^ would result in hampered or delayed docking to CXCR3A. Consistent with this hypothesis, we observed a delay in the initiation of calcium responses to CXCL10_(1–73)_ (Fig. 3B), which may be explained by delayed docking. Thus, the C-terminal residues of human CXCL10 may act analogous to mCXCL10, where Lys^71^-Arg^75^ are involved in CXCR3A activation resulting in downstream calcium signaling and chemotaxis (*vide supra*) [47]. Also, similar to mCXCL10 is the emerging notion that binding sites for GAG and CXCR3 are partially overlapping in human CXCL10. In addition, CXCL10_(1–73)_ has similar affinity for heparin and HS, whereas CXCL10_(1–77)_ has 6-times higher affinity for heparin compared to HS (Table 1). This may indicate that sulfation is less involved in GAG binding of CXCL10_(1–73)_, since heparin exhibits a greater degree of sulfation compared to HS (2.3 and 0.7 sulfates per disaccharide, respectively) [63].

Analogous to CD26-mediated N-terminally truncated CXCL10_(3–77)_ [34], we observed that the C-terminal truncation did not significantly modulate anti-angiogenic properties of CXCL10. Hence, proteolysis of CXCL10 in inflamed tissue would still favor angiostasis, thereby limiting additional leukocyte ingress in the respective epicenter of inflammation by preventing formation of new blood vessels. GAG-dependent angiostatic actions have been abundantly demonstrated for mCXCL10 [47, 48]. Given the limited expression of CXCR3 on HMVEC (median CXCR3 expression 24.5%; median mean fluorescence intensity [MFI] 731.5; Fig. S9F, G), GAG-dependent angiostasis may also play a role in anti-angiogenic effects induced by human CXCL10. Recently, CXCR3B was described to display atypical chemokine receptor (ACKR) features [81]. Furthermore, N-terminal truncation of CXCL10_(1–77)_ into CXCL10_(3–77)_ attenuated its ability to induce $\beta$-arrestin 1 recruitment towards CXCR3A and CXCR3B [81], implying that PTMs may also affect CXCR3B-mediated internalization of CXCL10. As such, CXCL10_(1–73)_ may proportionally be depleted less from the extracellular milieu by CXCR3B on HMVEC (in a $\beta$-arrestin-dependent or -independent manner) compared to intact CXCL10_(1–77)_. The reduced depletion of CXCL10_(1–73)_ may compensate for its reduced GAG binding and therefore CXCL10_(1–73)_ may exert a seemingly equal angiostatic effect as native CXCL10_(1–77)_. Thus, this mechanism may clarify the role of CXCR3B (as a scavenger [81]) in the GAG-dependent angiostatic effect of CXCL10. It may explain the retained angiostatic actions of CXCL10_(3–77)_ [34]. Hence, the angiostatic effect of CXCL10 may be mediated through competition with, and subsequent displacement of, growth factors to bind to GAGs [48], direct interaction with growth factors [82], and/or through cross-linking of GAGs and downstream signaling in endothelial cells [83]. Given that the CXCL10-derived peptide CXCL10_(56–77)_ exerted similar anti-angiogenic effects as full-length CXCL10_(1–77)_ [43], our findings may also imply that the C-terminal α-helical amino-acids (Ser^58^-Glu^71^; Fig. 9A) —rather than the endmost C-terminal coiled residues— are crucial for actions leading to downstream signaling resulting in angiostasis.

We are the first to describe the presence of C-terminally truncated CXCL10 in patient samples. We even detected a relatively higher abundancy of CXCL10_(1–73)_ compared to intact CXCL10_(1–77)_ in synovial fluids of RA patients, which further points towards a physiological role of the posttranslationally processed molecule in joint inflammation. In RA, synovial CXCL10 levels are highly increased, thereby establishing a chemotactic gradient from the blood towards the synovium [51]. As such, circulatory CXCL10 was even proposed to be a predictive marker for diagnosis of early RA, monitoring disease activity in established RA and predicting the response to anti-TNF-α treatment [84–87]. In this context, synovial fibroblasts were found to spontaneously secrete CXCL10, whereby simultaneous exposure to IFN-γ and TNF-α synergistically induced even more pronounced secretion of CXCL10 [52, 53]. Furthermore, enzymes reported to C-terminally truncate CXCL10, including furin, carboxypeptidase B, MMPs, and cathepsins, are all expressed in the synovium [88–94]. Proteolytic processing of CXCL10_(1–77)_ into CXCL10 proteoforms with reduced chemotactic activity but retained angiostatic features (e.g., CXCL10_(1–73)_) may be an elegant natural manner to dampen synovitis, whilst maintaining inhibitory effects on neoangiogenesis in the synovial niche [95].

## Conclusions

This study reveals that the four endmost C-terminal residues Lys^74^-Pro^77^ of CXCL10 are important for GAG binding, CXCR3A signaling, T lymphocyte chemotaxis, but dispensable for angiostasis. The upregulation of natural CXCL10_(1–73)_ in synovial fluids of patients with RA underscores the in vivo biological significance of this CXCL10 proteoform. In addition, the optimized SPPS approach to generate high quality synthetic CXCL10 paves the way towards research on other naturally occurring CXCL10 proteoforms. Given the validated role of CXCL10 in viral infection [37–41, 96, 97], tumor immunology [36, 98, 99], and autoimmune arthritis [51, 100], the balance between CXCL10 and its processing enzymes in inflamed tissues is pivotal for fine-tuning the effects of CXCL10 in (patho)physiological settings.

## Materials and methods

A Supplemental Experimental Procedure section is provided in the Supplemental Information.

### Cell cultures and reagents

Chinese hamster ovary (CHO) cells transfected with CXCR3A were cultured in Ham’s F-12 growth medium (Gibco; Thermo Fisher Scientific, Waltham, MA, USA) supplemented with 10% (*v/v*) heat-inactived fetal calf serum (FCS, Sigma-Aldrich, Saint Louis, MO, USA), 400 µg/ml G418 (Carl Roth, Karlsruhe, Germany), 1 mM sodium pyruvate (Gibco) and 0.12% (*v/v*) sodium bicarbonate (Gibco) [34]. Human microvascular endothelial cells (HMVEC; Cell Systems, Kirkland, WA, USA) were cultured in endothelial cell basal medium-2 (EBM-2: Lonza, Basel, Switzerland) supplemented with the EGM-2 MV SingleQuots kit (Lonza). For culturing of primary lymphocytes, PBMC were purified from buffy coats of healthy volunteers (Red Cross, Mechelen, Belgium) through gradient centrifugation, as previously described [101]. T lymphoblasts were generated through culturing mononuclear cells in 2 µg/mL PHA (Sigma-Aldrich) and 50 U/ml interleukin (IL)-2 (PeproTech, Rocky Hill, NJ, USA), as formerly described [34].

### Patients

Patients were previously described and recruited at the University Hospital of Leuven after providing their informed consent according to the ethical guidelines of the Declaration of Helsinki [102]. Briefly, synovial fluids were only collected in case joint aspiration was needed for treatment purposes. Synovial fluid were kept in BD vacutainer tubes containing ethylenediaminetetraacetic acid (EDTA) (BD Biosciences, East Rutherford, NJ). Synovial fluid was centrifuged at 400 g for 20 min at room temperature (RT). Thereafter, cell-free synovial fluid was collected and stored at -80 °C. The Ethics Committee of the University Hospital Leuven approved the experiments involving human subjects (ML1814, S59874 and S65508).

### Tandem mass spectrometry on synovial fluid samples of patients with RA

Relative abundancies of CXCL10_(1–77)_ and CXCL10_(1–73)_ were determined in synovial fluids of patients with RA using immunosorbent sample preparation and nano-scale liquid chromatography-tandem mass spectrometry (nano-LC–MS/MS) for proteoform analysis (ISTAMPA), as recently described [103].

### Chemical synthesis and purification of C-terminally truncated human CXCL10_(1–73)_

CXCL10_(1–73)_ was chemically synthesized based on *N*-(9-fluorenyl)methoxycarbonyl (Fmoc) chemistry using an Activo-P11 automated peptide synthesizer (Activotec, Cambridge, UK). A hydrophilic resin and specialized amino acid building blocks were used to ensure a successful SPPS.

### Surface plasmon resonance

Real-time binding kinetics of CXCL10 proteoforms with different GAGs (heparin, HS, and CS-A) were examined through SPR on a Biacore T200 instrument (Cytiva, Uppsala, Sweden) in a similar experimental set-up as previously described [104].

### Signal transduction assays

The potency of CXCL10_(1–77)_ and CXCL10_(1–73)_ to induce an increase of the intracellular calcium concentration was evaluated on CXCR3A-transfected CHO cells, as previously described [34, 105]. To determine phosphorylation of ERK1/2 and Akt upon chemokine treatment, 0.4 $\times$ 10^6^ CXCR3A-transfected CHO cells/ml or 60 000 HMVEC/ml (2 ml/well) were seeded in flat bottom 6-well plates (2.0 ml/well; TPP, Sigma-Aldrich) in supplemented Ham’s F-12 growth medium + 10% (*v/v*) FCS or EBM-2 cell culture medium, respectively. Upon overnight starvation in serum-free medium, cells were incubated with serum-free medium containing 0.5% (*w/v*) bovine serum albumin (BSA; endotoxin free, Sigma-Aldrich) for 15 min at 37 °C. Subsequently, cells were stimulated at 37 °C with CXCL10_(1–73)_ or CXCL10_(1–77)_ for 5 min (for CHO cells) or 15 min followed by 5 min stimulation with FGF-2 (for HMVEC). Signal transduction was terminated and pERK1/2 and pAkt was determined in the supernatant of cell lysates, as previously described [27, 105].

### Multiscreen chemotaxis assay with primary T lymphocytes

For the multiscreen chemotaxis assay (Millipore Corporation, Billerica, MA, USA), 96-well filter plates (5 µm pore-size) were either not pre-coated or pre-coated with bovine plasma FN (Gibco), human FN (BD Biosciences, San Jose, California, USA) or human type I collagen (Sigma-Aldrich) overnight. Primary T lymphocytes stimulated with PHA and IL-2 (2 $\times$ 10^5^, 100 µl/well) were resuspended in HBSS buffer (Gibco) containing 0.1% (*w/v*) BSA and 100 µM of the CD26 inhibitor sitagliptin (Januvia; Merck Sharpe & Dohme [MSD] Whitehouse Station, NJ, USA). T lymphocyte migration from the upper plate towards chemoattractant solution in the receiver plate was quantified via the ATP detection assay (Perkin Elmer, Waltham, MA). In parallel with the multiscreen assay, CXCR3 expression on PHA- and IL-2-activated T lymphocytes was evaluated through flow cytometry.

### CXCR3 internalization on primary T lymphocytes

Equal volumes of PHA- and IL-2 stimulated T cells (90 µl, 5.5 × 10^6^ cells/ml) were resuspended in PBS containing 100 µM sitagliptin (Januvia) and 2% FCS and stimulated with varying concentrations of CXCL10_(1–73)_ and CXCL10_(1–77)_ for 10 min at 37 °C. After incubation, cells were put on ice and washed once with ice-cold flow cytometry buffer. After centrifugation at 4 °C for 5 min at 300* g*, cells were resuspended in PBS. Internalization of CXCR3 was analyzed with flow cytometry in a similar manner as described to evaluate the CXCR3 expression on T lymphocytes used for multiscreen chemotaxis assays (vide supra). The relative surface expression of CXCR3 was calculated as a percentage relative to medium treated cells.

### xCELLigence chemotaxis assay for HMVEC

The xCELLigence® real-time cell analyzer double plate (RTCA-DP) system (ACEA Biosciences, Inc.; San Diego, CA, USA) was utilized to evaluate HMVEC migration. Briefly, 160 µl of control medium (i.e., EBM-2 medium containing 0.4% [*v/v*] FCS) with or without 30 ng/ml FGF-2 was added to the lower chamber of a cell invasion/migration (CIM) plate in the presence or absence of CXCL10_(1–77)_ or CXCL10_(1–73)_ at varying concentrations (1.2 nM, 12 nM, 120 nM, or 360 nM). Upon chamber assembly and addition of HMVEC (4 $\times$ 10^4^ cells/well) to the upper compartment, alterations in electrical impedance were measured and converted into cell indices. To compare HMVEC migration induced by CXCL10_(1–73)_ and CXCL10_(1–77)_ relative to control medium or FGF-2-treated cells at 12 h, cell indices measured upon incubation with control medium were set to 100%.

### In vitro toxicity assay

HMVEC were seeded at 8 $\times$ 10^3^ cells/well in MCDB131 medium + 3% (*v/v*) FCS in a black, clear bottom 96-well plate (Greiner Bio-one, Kremsmünster, Austria) coated with 0.1% (*v/v*) gelatin in PBS. Following overnight incubation (37 °C, 5% CO_2_), cells were washed and incubated with control medium (*i.e*., MCDB131 supplemented with 0.4% (*v/v*) FCS) in the presence or absence of CXCL10_(1–77)_ or CXCL10_(1–73)_ for 30 h at 37 °C and 5% CO_2_. Toxicity of the CXCL10 proteoforms was evaluated using the LIVE/DEAD Viability/Cytotoxicity Kit for mammalian cells (Invitrogen, Thermo Fisher Scientific) according to the manufacturer’s instructions.

### Proliferation assay

HMVEC were seeded at 5 $\times$ 10^3^ cells/well in EBM-2 cell culture medium in a flat bottom 96-well plate (Greiner Bio-One). After overnight settling of the cells, cells were starved for 4 h in EBM-2 + 1% [*v/v*] FCS. After starvation, cells were stimulated with FGF-2 (10 ng/ml) alone or in combination with CXCL10 proteoforms. The luminescence ATPlite assay (Perkin Elmer) was used according to the manufacturer’s instruction after 4 days to assess proliferation.

### Scratch wound assay

HMVEC were seeded at 15 $\times$ 10^3^ cells/well in EBM-2 cell culture medium in an IncuCyte ImageLock 96-well plate (Essen Bioscience; Newark, UK) coated with 0.1% (*v/v*) gelatin in PBS. Following overnight incubation (37 °C, 5% CO_2_), 700 – 800 µm wide wounds were simultaneously created in the endothelial monolayers of all wells using an IncuCyte 96-well Woundmaker Tool (Essen Bioscience). Cells were washed twice in basal EBM-2 medium and incubated with control medium (*i.e*., EBM-2 + 1% [*v/v*] FCS), 1 ng/ml FGF-2 or CXCL10_(1–77)_ or CXCL10_(1–73)_ in the presence or absence of 1 ng/ml FGF-2. HMVEC were monitored for 17 h in the IncuCyte S3 Live-Cell Analysis System to determine wound confluence and relative wound density.

### Spheroid sprouting assay

Single spheroids were formed in hanging droplets, collected and distributed over a clear flat bottom 96-well plate in a methylcellulose/collagen type I suspension as previously described [104]. Spheroids were left untreated (addition of control medium, i.e. EBM-2 $+$ 3% [*v/v*] FCS) or were incubated with 12 nM or 120 nM CXCL10_(1–77)_ or CXCL10_(1–73)_ at 37 °C and 5% CO_2_ for 15 min prior to the addition of 10 ng/ml FGF-2 in EBM-2 $+$ 3% (*v/v*) FCS. Following 17 h incubation at 37 °C and 5% CO_2_, sprouting of the spheroids was evaluated with bright field imaging through a 10 $\times$ objective on an inverted Axiovert 200 M microscope (Carl Zeiss Microscopy GmbH, Oberkochen, Germany). The average number of sprouts per spheroid and the average cumulative sprout length per spheroid for each well was calculated using ImageJ software (NIH; Bethesda, Maryland, USA).

### In vivo cell migration assay

Drinking water of 8-week old Naval Medical Research Institute (NMRI) mice was supplemented with 1.667 mg/ml of CD26 inhibitor sitagliptin (Januvia; 0.22 $\mu$m filtered) for 72 h prior to an IP injection of 10 µg recombinant CXCL10_(1–77)_ or synthetic CXCL10_(1–73)_. An estimated consumption of 10 mg/day of sitagliptin for two days was previously described to reduce the residual CD26 activity in the mouse plasma to 26% as compared to plasma of naïve untreated mice [106]. Drinking volume was monitored daily. A *Limulus* amoebocyte lysate assay (Cambrex Corporation, East Rutherford, NJ, USA) showed that CXCL10_(1–73)_ and CXCL10_(1–77)_ stock solutions contained very low endotoxin levels (< 0.06 pg LPS/µg of chemokine). Mice were euthanized with a subcutaneous injection of 300 µl Dolethal (pentobarbital; 200 mg/ml; Vétoquinol, Aartselaar, Belgium) 16 h after chemokine injection, and peritoneal cavities were washed with 5 ml PBS supplemented with 2% (*v/v*) FCS and 20 U/ml heparin (Leo Pharma, Amsterdam, the Netherlands). Cells were analyzed through flow cytometry.

### CD26 activity assay

After collection of cells for analysis by flow cytometry, peritoneal lavage fluids of NMRI mice were centrifuged at 300 g for 10 min at 4 °C and supernatant was collected and stored at -20 °C. In a flat bottom 96-well plate, lavage fluids (1/2 diluted; 100 µl) were incubated with 500 µM Gly-Pro-*p*-nitroanilide substrate (Sigma-Aldrich) in 200 mM Tris–HCl buffer (pH 8.3) to determine the soluble CD26 enzymatic activity. Given that sitagliptin is a competitive inhibitor of CD26 and the peritoneal lavage fluids were ½ diluted, the inhibition of the soluble CD26 activity in the peritoneal fluid is underestimated in the CD26 activity assay. Therefore, a calibration curve to estimate the residual CD26 activity was generated, as previously described [107] (Fig. S7B).

### Vascular permeability assay

HMVEC were seeded (10 000 cells/well) on gelatin-coated membranes with 0.4 µm pores and 6.5 mm diameter (Transwell; Corning, New York) and were grown to confluence in EBM-2 cell culture medium. After starving the cells overnight in EBM-2 + 1% [*v/v*] FCS, cells were treated with 100 ng/ml VEGF (Biolegend; San Diego, California, USA) alone or in combination with 360 nM CXCL10_(1–77)_ or CXCL10_(1–73)_ in the upper chambers for 3 h. Afterwards, leakage of 1 mg/ml fluorescein isothiocyanate (FITC)-conjugated dextran (70 kDa; Sigma-Aldrich) from the top to the bottom compartment was used to calculate permeability. To check whether cell confluence was obtained, we seeded a 96-well plate in parallel at equal cell density (well surface is identical to the surface of a transwell insert) and we performed confocal microscopy on the inserts.

### Evaluation of expression of lymphocyte adhesion molecules and junctions on endothelial cells

HMVEC were seeded (140 000 cells/well) in a flat bottom 6-well plate in EBM-2 cell culture medium. Upon adherence, HMVEC were treated for 48 h at 37 °C and 5% CO_2_ with EBM-2 medium + 3% FCS (CO), a combination of 100 ng/ml TNF-α (Peprotech) and 100 ng/ml IFN-γ (Peprotech) to induce lymphocyte adhesion molecules, or CXCL10_(1–77)_ or CXCL10_(1–73)_ at 120 nM or 360 nM. Thereafter, medium was removed and cells were washed in cold PBS. Cells were detached using cell scrapers (Sarstedt, Darmstadt, Germany) in 100 µl cold PBS to avoid trypsinization. Cells were transferred to FACS tubes, stained and analyzed.

### Statistical analysis

GraphPad Prism software 10.0.3 (San Diego, California, USA) and MATLAB® (Natick, Massachusetts, USA) were used for data analysis. Shapiro–Wilk test was used to assess if data were normally distributed. A Mann–Whitney U test or Kruskal–Wallis test with Dunn’s multiple comparison correction was used for data that were not normally distributed (displayed as median ± IQR). Unpaired t-tests were used when the data exhibited normal distribution (displayed as mean ± SEM).

### Supplementary Information

**Additional file 1: Figure S1.** (related to Figure 1) shows the experimental optimization of the solid phase peptide synthesis of human CXCL10_(1-73)_. **Figure S2.** (related to Figure 2) demonstrates the fitted curves on SPR sensorgrams for interactions of heparin, heparan sulfate and chondroitin sulfate via the 1:1 binding model with mass transfer correction. **Figure S3.** (related to Figure 3H-I) shows the gating strategy for evaluation of CXCR3 expression on primary T lymphocytes stimulated with phytohemagglutinin (PHA) and IL-2. **Figure S4.** (related to Figure 3) shows that CXCL10_(1-73)_ also induces significantly less T lymphocyte migration through membranes coated with different extracellular matrix proteins compared to CXCL10_(1-77)_. **Figure S5.** (related to Figure 5) shows high quality images of the wound borders and area with reduced wound density upon treatment with CXCL10_(1-77)_ and CXCL10_(1-73)_. **Figure S6.** (related to Figure 5) shows that FGF-2-induced HMVEC migration and invasion is inhibited by CXCL10_(1-77)_ and CXCL10_(1-73)_ at 360 nM. **Figure S7.** (related to Figure 7) shows that the average consumption of sitagliptin per mouse is equivalent for groups that were treated with vehicle, CXCL10_(1-77)_ or CXCL10_(1-73)_. **Figure S8.** (related to Figure 7) shows that trends towards increased ingress of T cells, CD4^+^ T cells, NKT cells and B cells and their activated CXCR3^+^ subsets were found for mice treated with CXCL10_(1-77)_, but not for those receiving CXCL10_(1-73)_. **Figure S9.** (related to Figure 5-8) shows that CXCL10_(1-77)_ and CXCL10_(1-73)_ do not affect the expression of adherence junction vascular endothelial (VE)-cadherin nor tight junction zona occludens 1 (ZO-1). This figure also shows the CXCR3 expression by HMVEC and CXCR3A-transfected CHO cells in culture. **Table S1.** (related to Figure 3E and Figure 3H-I) shows the list of antibodies used for immunophenotyping of CXCR3-expressing primary T lymphocytes stimulated with PHA and IL-2. **Table S2.** (related to Figure 7) shows a list of the antibodies used for immunophenotyping of peritoneal lymphoid cells harvested after peritoneal lavages from sitagliptin-treated NMRI mice. **Table S3.** (related to Figure 8) shows a list of the antibodies used for immunophenotyping of endothelial cells to evaluate lymphocyte adhesion molecules, adherence and tight junctions.
