# Supplementary material for: Natural carboxyterminal truncation of human CXCL10 attenuates glycosaminoglycan binding, CXCR3A signaling and lymphocyte chemotaxis, while retaining angiostatic activity
Source: Cell Commun Signal. 2024 Feb 2;22:94. doi: 10.1186/s12964-023-01453-1 (PMC10835923; doi:10.1186/s12964-023-01453-1)
Supplement: Supplementary file 1 — Additional file 1: Figure S1. (related to Figure 1) shows the experimental optimization of the solid phase peptide synthesis of human CXCL10(1-73). Figure S2. (related to Figure 2) demonstrates the fitted curves on SPR sensorgrams for interactions of heparin, heparan sulfate and chondroitin sulfate via the 1:1 binding model with mass transfer correction. Figure S3. (related to Figure 3H-I) shows the gating strategy for evaluation of CXCR3 expression on primary T lymphocytes stimulated with phytohemagglutinin (PHA) and IL-2. Figure S4. (related to Figure 3) shows that CXCL10(1-73) also induces significantly less T lymphocyte migration through membranes coated with different extracellular matrix proteins compared to CXCL10(1-77). Figure S5. (related to Figure 5) shows high quality images of the wound borders and area with reduced wound density upon treatment with CXCL10(1-77) and CXCL10(1-73). Figure S6. (related to Figure 5) shows that FGF-2-induced HMVEC migration and invasion is inhibited by CXCL10(1-77) and CXCL10(1-73) at 360 nM. Figure S7. (related to Figure 7) shows that the average consumption of sitagliptin per mouse is equivalent for groups that were treated with vehicle, CXCL10(1-77) or CXCL10(1-73). Figure S8. (related to Figure 7) shows that trends towards increased ingress of T cells, CD4+ T cells, NKT cells and B cells and their activated CXCR3+ subsets were found for mice treated with CXCL10(1-77), but not for those receiving CXCL10(1-73). Figure S9. (related to Figure 5-8) shows that CXCL10(1-77) and CXCL10(1-73) do not affect the expression of adherence junction vascular endothelial (VE)-cadherin nor tight junction zona occludens 1 (ZO-1). This figure also shows the CXCR3 expression by HMVEC and CXCR3A-transfected CHO cells in culture. Table S1. (related to Figure 3E and Figure 3H-I) shows the list of antibodies used for immunophenotyping of CXCR3-expressing primary T lymphocytes stimulated with PHA and IL-2. Table S2. (related to Figure 7) shows a l [file 12964_2023_1453_MOESM1_ESM.pdf]

SUPPLEMENTAL INFORMATION

Natural carboxyterminal truncation of human CXCL10 attenuates glycosaminoglycan binding, CXCR3A signaling and lymphocyte chemotaxis, while retaining angiostatic activity

Luna Dillemans, Karen Yu, Alexandra De Zutter, Sam Noppen, Mieke Gouwy, Nele Berghmans, Lisa Verhallen, Mirre De Bondt, Lotte Vanbrabant, Stef Brusselmans, Erik Martens, Dominique Schols, Patrick Verschueren, Mette Rosenkilde, Pedro Elias Marques, Sofie Struyf, and Paul Proost

INVENTORY OF THE SUPPLEMENTAL INFORMATION

- **Figure S1** (related to **Figure 1**) shows the experimental optimization of the solid phase peptide synthesis of human CXCL10$_{(1-73)}$.
- **Figure S2** (related to **Figure 2**) demonstrates the fitted curves on SPR sensorgrams for interactions of heparin, heparan sulfate and chondroitin sulfate via the 1:1 binding model with mass transfer correction.
- **Figure S3** (related to **Figure 3H-I**) shows the gating strategy for evaluation of CXCR3 expression on primary T lymphocytes stimulated with phytohemagglutinin (PHA) and IL-2.
- **Figure S4** (related to **Figure 3**) shows that CXCL10$_{(1-73)}$ also induces significantly less T lymphocyte migration through membranes coated with different extracellular matrix proteins compared to CXCL10$_{(1-77)}$.
- **Figure S5** (related to **Figure 5**) shows high quality images of the wound borders and area with reduced wound density upon treatment with CXCL10$_{(1-77)}$ and CXCL10$_{(1-73)}$.
- **Figure S6** (related to **Figure 5**) shows that FGF-2-induced HMVEC migration and invasion is inhibited by CXCL10$_{(1-77)}$ and CXCL10$_{(1-73)}$ at 360 nM.
- **Figure S7** (related to **Figure 7**) shows that the average consumption of sitagliptin per mouse is equivalent for groups that were treated with vehicle, CXCL10$_{(1-77)}$ or CXCL10$_{(1-73)}$.
- **Figure S8** (related to **Figure 7**) shows that trends towards increased ingress of T cells, CD4$^+$ T cells, NKT cells and B cells and their activated CXCR3$^+$ subsets were found for mice treated with CXCL10$_{(1-77)}$, but not for those receiving CXCL10$_{(1-73)}$.
- **Figure S9** (related to **Figure 5-8**) shows that CXCL10$_{(1-77)}$ and CXCL10$_{(1-73)}$ do not affect the expression of adherence junction vascular endothelial (VE)-cadherin nor tight junction zona occludens 1 (ZO-1). This figure also shows the CXCR3 expression by HMVEC and CXCR3A-transfected CHO cells in culture.
- **Table S1** (related to **Figure 3E** and **Figure 3H-I**) shows the list of antibodies used for immunophenotyping of CXCR3-expressing primary T lymphocytes stimulated with PHA and IL-2.
- **Table S2** (related to **Figure 7**) shows a list of the antibodies used for immunophenotyping of peritoneal lymphoid cells harvested after peritoneal lavages from sitagliptin-treated NMRI mice.
- **Table S3** (related to **Figure 8**) shows a list of the antibodies used for immunophenotyping of endothelial cells to evaluate lymphocyte adhesion molecules, adherence and tight junctions.
- Supplemental Experimental Procedures
- Supplemental References

SUPPLEMENTARY FIGURES AND TABLES

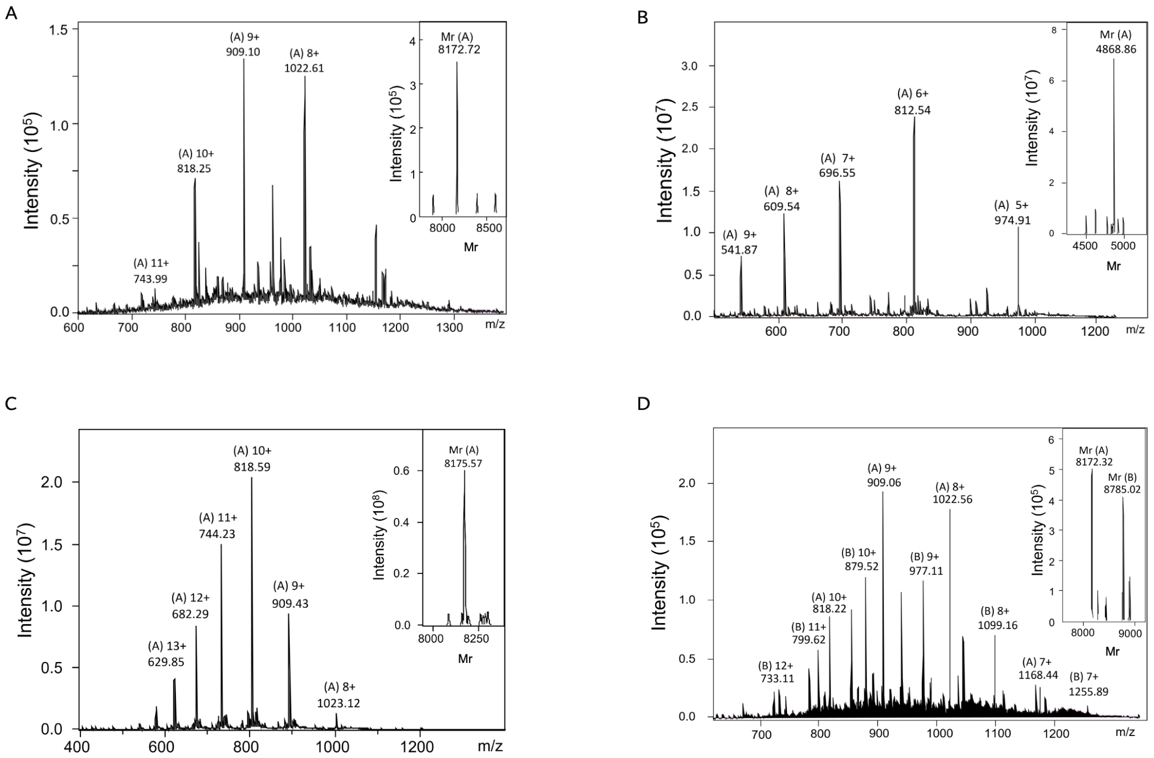

E

Synthetic CXCL10 proteoform	Theoretical Mr	Experimental Mr
$CXCL10_{(1-73)}$ (oxidized state)	8173.65	8172.72 (Fig. S1A)
Acetylated $CXCL10_{(31-73)}$ (reduced state)	4870.78	4868.86 (Fig. S1B)
$CXCL10_{(1-73)}$ (reduced state)	8177.69	8175.57 (Fig. S1C)
$CXCL10_{(1-73)}$ coupled by two glutathione residues	8788.31	8785.02 (Fig. S1D)

SUPPLEMENTARY FIGURE S1. Averaged mass spectrum for molecules present in the CXCL10-containing fractions after RP-HPLC of human synthetic $CXCL10_{(1-73)}$ generated via SPPS. The intensity of the detected ions with their specific mass/charge (m/z) ratio is shown along with the relative molecular mass (Mr) of the proteins corresponding with these ions (insets on the right). SPPS was performed (**A**) using Fmoc-Ser(But)-Wang resin and HATU and DIEA as a coupling system or (**B**) using a H-Ser(OtBu)-HMPB NovaPEG resin, HCTU and NMM as a coupling system and four pseudoprolines at positions Arg^{5}-Thr^{6}, Ile^{12}-Ser^{13}, Ala^{43}-Thr^{44} and Val^{68}-Ser^{69}. (**C**) SPPS was performed using the same conditions as indicated in panel B but with the additional use of a specific dipeptide building block at Ile^{30}-Pro^{31}. (**D**) Folding of synthetic $CXCL10_{(1-73)}$ was initially performed through incubation with 150 nM Tris, 3 mM EDTA, 0.3 mM reduced glutathione (GSH) 3 mM oxidized glutathione (GSSG), and 1 M guanidine hydrochloride (pH 8.6) as folding buffer under continuous rotation for 5 h. (**E**) The experimental Mr of the synthetic (un)folded protein is compared with the theoretical Mr of the CXCL10 proteoforms. DIEA, di-isopropylethylamine; Fmoc, 9-fluorenylmethoxycarbonyl; HATU, 2-(1H-7-azabenzotriazol-1-yl)-1,1,3,3-tetramethyluronium hexafluorophosphate; HCTU, O-(1H-6-chloro-benzotriazole-1-yl)-1,1,3,3-tetramethyluronium hexafluorophosphate; HMPB, 4-(4-hydroxymethyl-3-methoxyphenoxy)butyric acid; NMM, 4-Methylmorpholine; NMP, N-Methyl-2-pyrrolidone; OtBu, tert-butyl ester; PEG, polyethylene glycol.

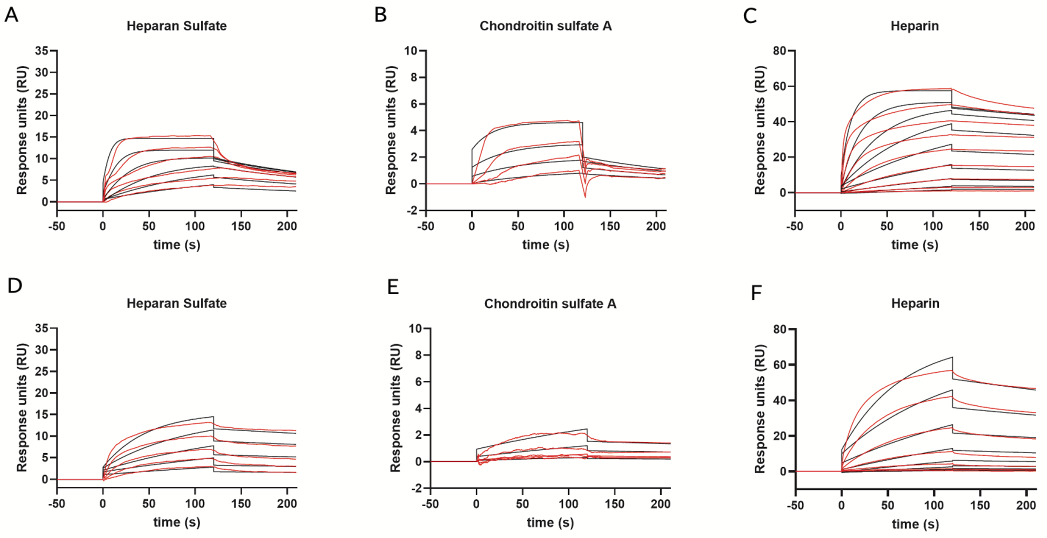

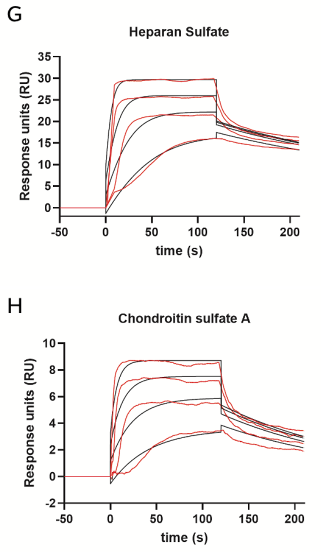

I

		χ²-values				
**Chemokine**	**GAG**	**1**	**2**	**3**	**4**	**5**
**CXCL4**	**HS**	2.78	1.99	1.25		
	**CS-A**	0.22	0.17	0.15		
**CXCL10$_{(1-77)}$**	**H**	11.9	8.33	6.06	5.05	4.86
	**HS**	0.82	0.30	0.13	0.117	
	**CS-A**	0.09	0.06	0.03	0.03	
**CXCL10$_{(1-73)}$**	**H**	4.56	2.39	2.72	2.23	
	**HS**	0.30	0.25	0.07	0.108	
	**CS-A**	0.26	0.01	0.03		

SUPPLEMENTARY FIGURE S2. Representative plots of SPR analysis and fitting for 1:1 binding model with mass transfer correction. Fitted curves on SPR sensorgrams for interactions of heparin, heparan sulfate and chondroitin sulfate with (**A-C**) CXCL10$_{(1-77)}$, (**D-F**) CXCL10$_{(1-73)}$, and (**G-H**) CXCL4. For interaction with heparan sulfate and chondroitin sulfate A, CXCL10$_{(1-77)}$ (250, 125, 62.5, 31.3, 15.6, 7.81, 3.91 and 1.95 nM), CXCL10$_{(1-73)}$ (1000, 500, 250, 125, 62.5, 31.3, 15.6 and 7.81 nM) or CXCL4 (250, 125, 62.5, 31.3, 15.6, 7.81, 3.91 and 1.95 nM) were sent over a neutravidin-coated CM4 Biacore chip surface. In addition, for interaction with heparin, CXCL10$_{(1-77)}$ (100, 50, 25, 12.5, 6.25, 3.13, 1.56, 0.781, 0.391 and 0.195 nM) or CXCL10$_{(1-73)}$ (360, 180, 90, 45, 22.5, 11.3, 5.63 and 2.81 nM) were passed over the heparin-bound neutravidin-coated CM4 Biacore chip surface. Experimental data (red lines) overlaid with fitted data (black lines) are displayed. (**I**) Qualitative interpretation of fitted curves on the sensorgrams was performed based on χ^2 to evaluate fitting quality. χ^2-values for the fits for each of the different experiments are displayed (n = 3-5).

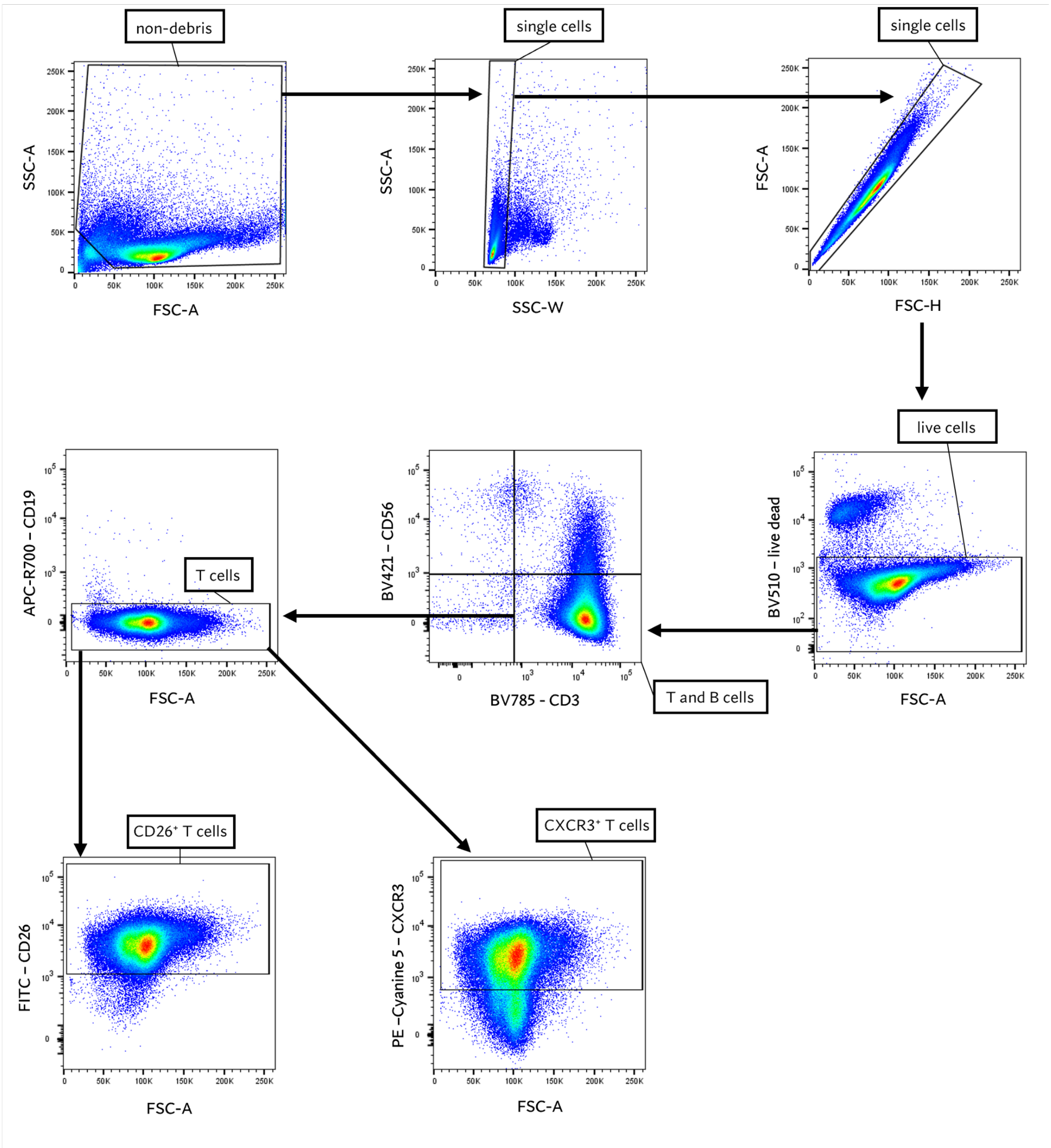

SUPPLEMENTARY FIGURE S3. Gating strategy for flow cytometry of primary T lymphocytes stimulated with PHA and IL-2. Cells were stained with fluorophore-conjugated monoclonal antibodies as indicated in Suppl. Table 1. First, debris and RBC were gated out based on size, followed by excluding cell doublets and dead cells (positive for live-dead [L/D] staining; BV510 Zombie Aqua). Then, leukocytes (i.e., $CD45^+$ cells) were selected from the remaining live cells. From the $CD45^+$ population, T cells ($CD3^+$ $NK1.1^-$ $CD19^-$) were selected. CXCR3 and CD26 were subsequently gated to select the activated $CXCR3^+$ T cell subpopulation and $CD26^+$ T cell subset, respectively.

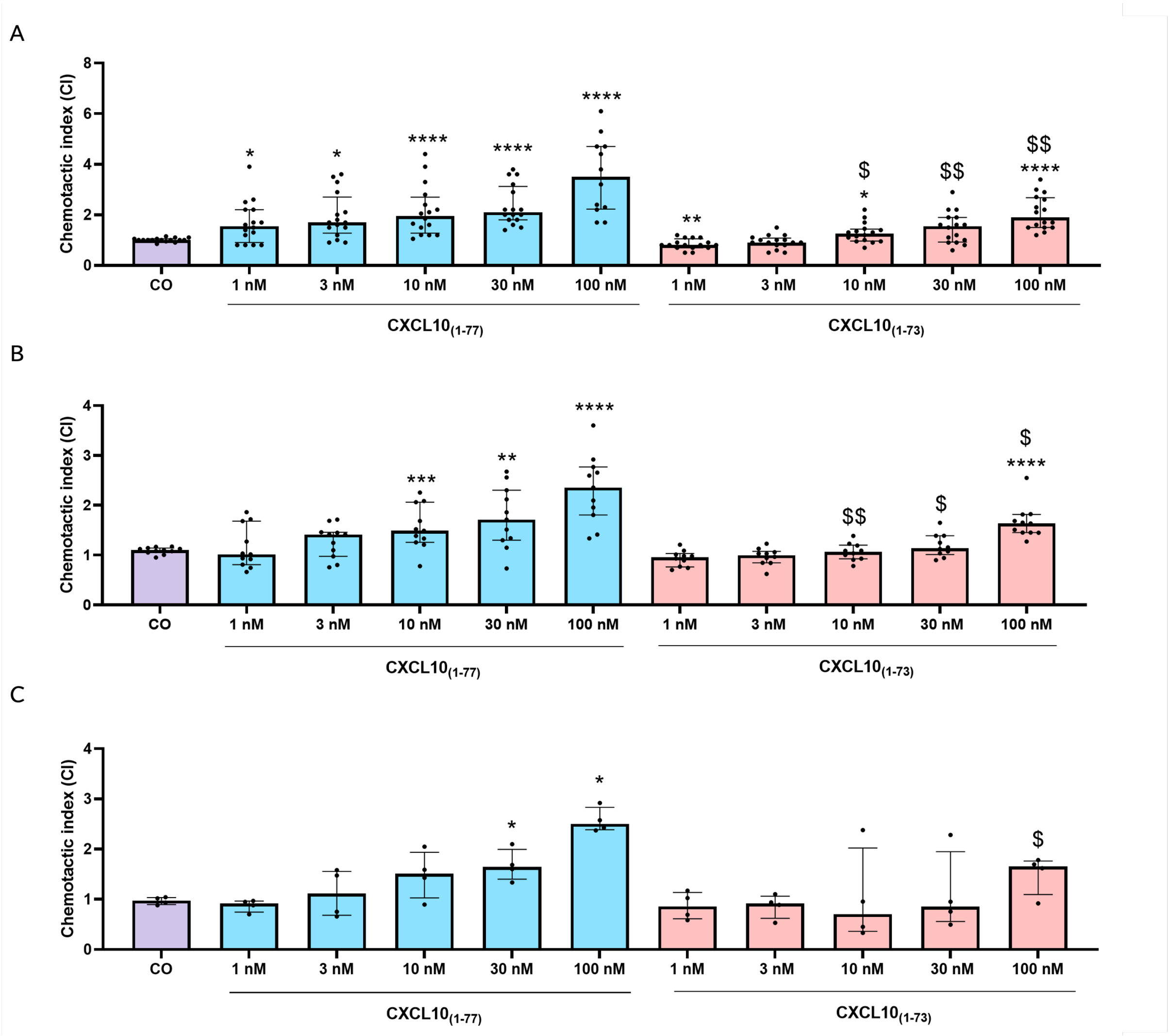

SUPPLEMENTARY FIGURE S4. Migration of CXCR3$^+$ T lymphocytes stimulated with PHA and IL-2 towards intact CXCL10$_{(1-77)}$ or C-terminally truncated CXCL10$_{(1-73)}$. Chemotactic index (CI) showing migration of PHA- and IL-2-stimulated T lymphocytes through membranes coated with (**A**) bovine FN, (**B**) human FN, or (**C**) human type I collagen after treatment with medium (HBSS + 0.1% BSA) as control condition (CO) or serial dilution of CXCL10$_{(1-77)}$ (100 nM to 1 nM) or CXCL10$_{(1-73)}$ (100 nM to 1 nM). Results are shown as median (± IQR) of 3 to 4 independent experiments with different cell preparations: 16 for bovine FN, 11 for human FN and 4 for collagen I. Statistically significant migration of CXCL10$_{(1-77)}$ or CXCL10$_{(1-73)}$ compared to CO were determined by Mann-Whitney U test (* $p \leq 0.05$, **, $p \leq 0.01$, *** $p \leq 0.001$, **** $p \leq 0.0001$). Statistically significant migration of CXCL10$_{(1-77)}$ compared to CXCL10$_{(1-73)}$ was also determined through a Mann-Whitney U test ($ $p \leq 0.05$, $$, $p \leq 0.01$). No significant differences were detected between similar concentrations of CXCL10 proteoforms in Millipore chemotaxis assays with membranes coated with human FN or human type I collagen.

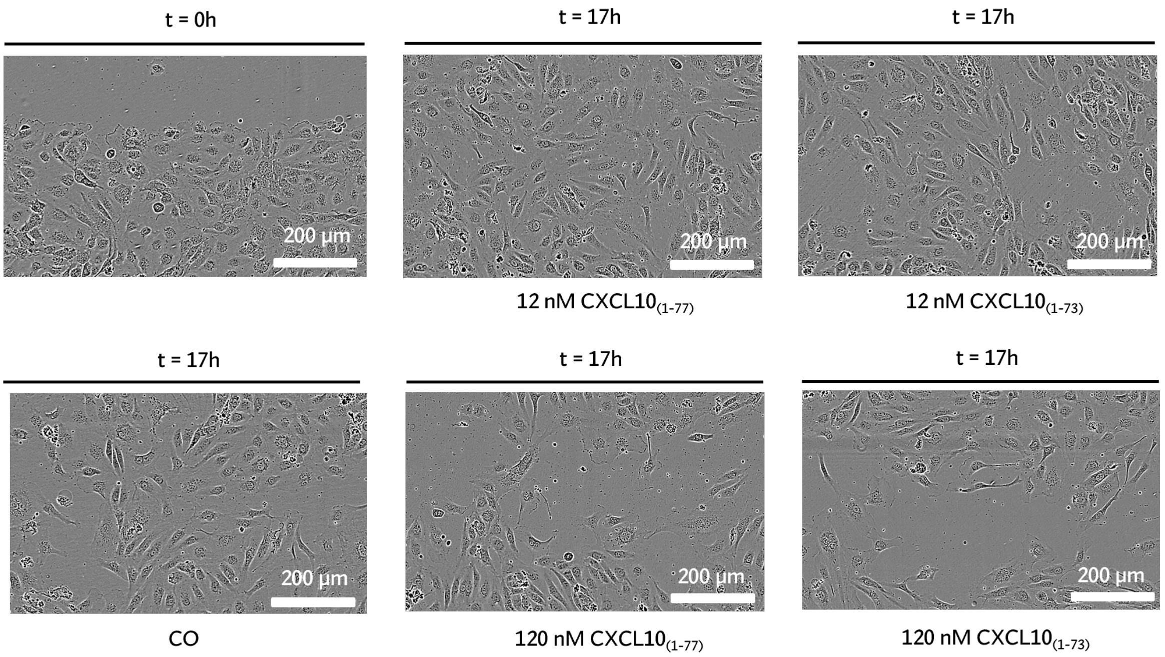

SUPPLEMENTARY FIGURE S5. Inhibition of spontaneous HMVEC migration and invasion by intact CXCL10$_{(1-77)}$ or C-terminally truncated CXCL10$_{(1-73)}$. After creating a scratch wound, spontaneous HMVEC migration and invasion was monitored for 17 h in EBM-2 + 1 % FCS (CO) in the presence or absence of CXCL10$_{(1-77)}$ or CXCL10$_{(1-73)}$ using the IncuCyte S3 Live-Cell Analysis System. Visualization of the wound borders at 0 h or 17 h using IncuCyte time-lapsed microscopy pictures of HMVEC stimulated with EBM-2 + 1% FCS (CO), CXCL10$_{(1-77)}$ or CXCL10$_{(1-73)}$ at 12 nM and 120 nM. Scale bar = 200 µm.

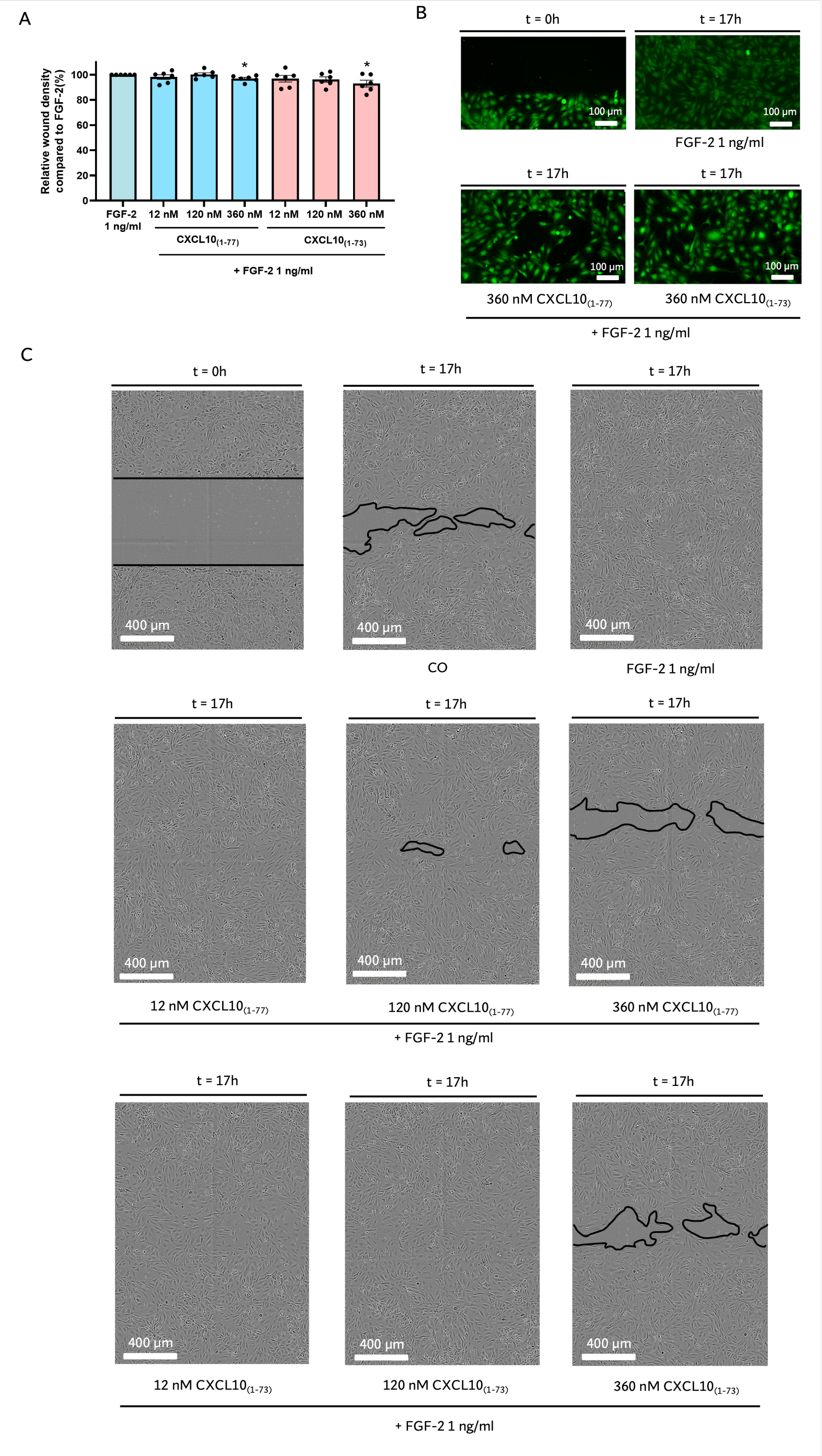
A
Relative wound density compared to FGF-2(%)
0
20
40
60
80
100
*
*
FGF-2 1 ng/ml
12 nM
120 nM
360 nM
12 nM
120 nM
360 nM
CXCL10(1-77)
CXCL10(1-73)
+ FGF-2 1 ng/ml
B
t = 0h
t = 17h
100 µm
FGF-2 1 ng/ml
t = 17h
t = 17h
360 nM CXCL10(1-77)
360 nM CXCL10(1-73)
+ FGF-2 1 ng/ml
C
t = 0h
t = 17h
t = 17h
400 µm
CO
FGF-2 1 ng/ml
12 nM CXCL10(1-77)
120 nM CXCL10(1-77)
360 nM CXCL10(1-77)
+ FGF-2 1 ng/ml
12 nM CXCL10(1-73)
120 nM CXCL10(1-73)
360 nM CXCL10(1-73)
+ FGF-2 1 ng/ml

SUPPLEMENTARY FIGURE S6. Inhibition of FGF-2-induced HMVEC migration and invasion by intact CXCL10$_{(1-77)}$ or C-terminally truncated CXCL10$_{(1-73)}$. After creating a scratch wound, FGF-2-induced HMVEC migration and invasion was monitored for 17 h in EBM-2 + 1 % FCS (CO) in the presence or absence of CXCL10$_{(1-77)}$ or CXCL10$_{(1-73)}$ using the IncuCyte S3 Live-Cell Analysis System. The basic analyzer unit of the Incucyte S3 2017 Software was used to calculate relative wound density. (**A**) Percentages of relative wound density compared to FGF-2-treated cells were represented in bar plots. The data are displayed as mean (± SEM) of 6 independent experiments. Unpaired t-test was used to compare differences in relative wound density compared to FGF-2-treated cells (* $p \leq 0.05$). Representative images of the full wound area and wound borders at 0 h or 17 h using (**B**) immunofluorescence microscopy after calcein staining (scale bar = 100 µm) or (**C**) IncuCyte time-lapsed microscopy pictures (scale bar = 400 µm) of HMVEC stimulated with 1 ng/ml FGF-2 alone or in combination with CXCL10$_{(1-77)}$ or CXCL10$_{(1-73)}$ at 12 nM, 120 nM or 360 nM.

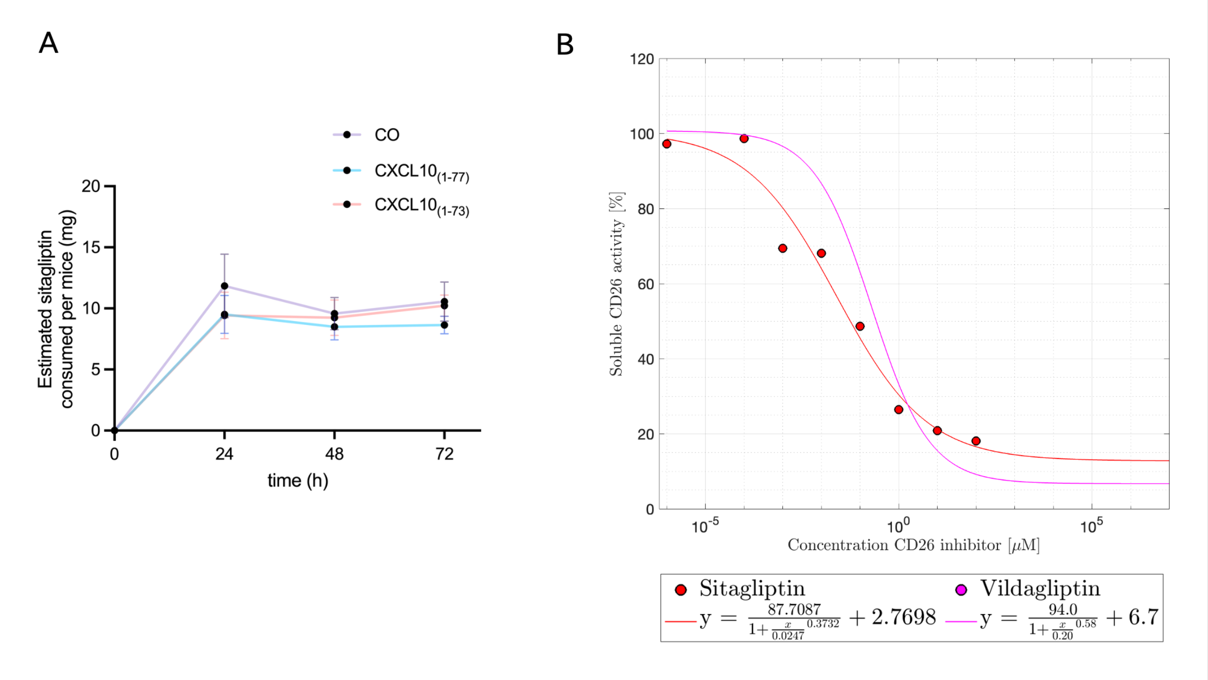

SUPPLEMENTARY FIGURE S7. Sitagliptin treatment of NMRI mice and residual CD26 activity. (**A**) The estimated average consumption of sitagliptin per mouse over 72 h is 10 mg/day. Values represent the median sitagliptin consumption per mouse (mg) ± IQR of 4 independent experiments (n = 16 per group). Statistical analysis was performed using Mann-Whitney U test (no significant differences between groups). (**B**) Calibration curve of the soluble CD26 activity (expressed as a percentage compared to a naïve sample) in function of the concentration of CD26 inhibitor (μM). Calibration curve for vildagliptin (pink) was plotted based on the equation provided by Matheeussen *et al.* [1]. Calibration curve for sitagliptin (red) was experimentally determined. A serial dilution of sitagliptin was added to peritoneal fluids of naïve untreated mice. CD26 activity was measured for each sample of the serial dilution (in duplicate) and expressed as a percentage compared to a naïve sample (which contained neither endogenous nor exogenously added sitagliptin) in function of the sitagliptin concentration. The coefficients were obtained from non-linear least squares fitting of the equation provided by Matheeussen *et al.* [1] (i.e., $y = \frac{Yrange}{1+\frac{x}{IC50}^{slope}} + background$) to the experimental results.

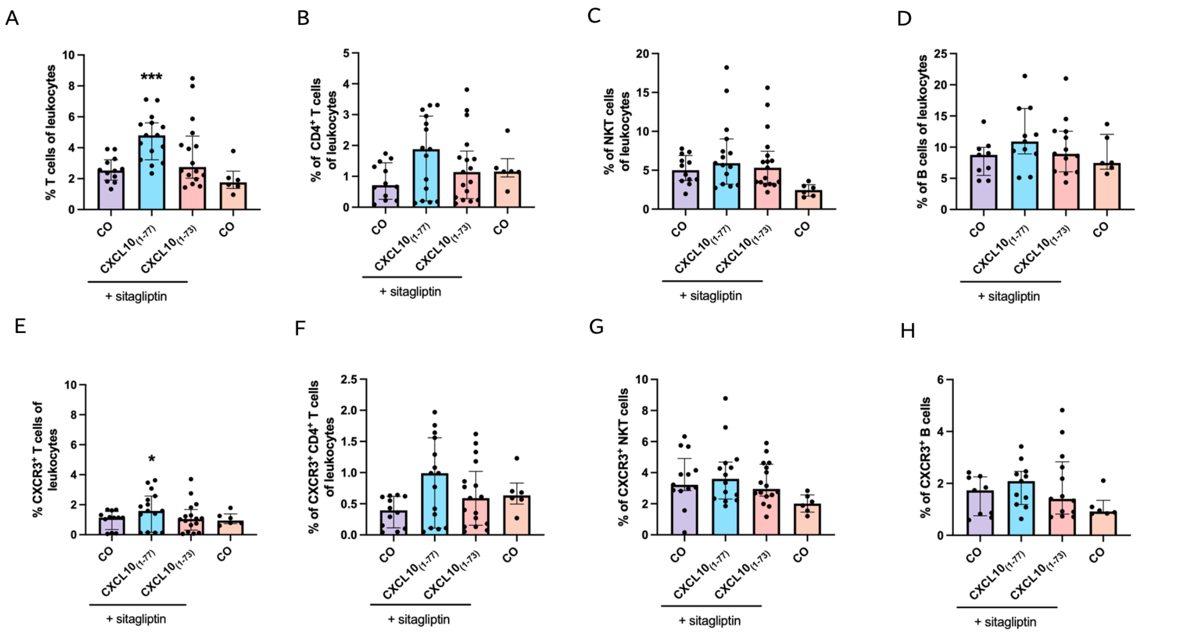

SUPPLEMENTARY FIGURE S8. Migration of CXCR3$^+$ lymphoid cells upon intraperitoneal injection of 10 µg intact CXCL10$_{(1-77)}$ or C-terminally truncated CXCL10$_{(1-73)}$ in sitagliptin-treated NMRI mice. Proportions relative to total leukocytes (CD45$^+$ cells) of (**A**) T cells (gated as CD3$^+$ NK1.1$^-$), (**B**) CD4$^+$ helper T cells (gated as CD3$^+$ NK1.1$^-$ CD4$^+$), (**C**) NKT cells (gated as CD3$^+$ NK1.1$^+$), and (**D**) B cells (gated as CD3$^-$ NK1.1$^-$ CD19$^+$). For activated cells, proportions relative to total leukocytes (CD45$^+$ cells) of (**E**) activated CXCR3$^+$ T cells (gated as CD3$^+$ NK1.1$^-$ CXCR3$^+$), (**F**) activated CXCR3$^+$ CD4$^+$ T cells (gated as CD3$^+$ NK1.1$^-$ CD4$^+$ CXCR3$^+$), (**G**) CXCR3$^+$ NKT cells (gated as CD3$^+$ NK1.1$^+$ CXCR3$^+$), and (**H**) CXCR3$^+$ B cells (gated as CD3$^-$ NK1.1$^-$ CD19$^+$ CXCR3$^+$). Each symbol represents an individual mouse ($n \geq 6$ per group). Four independent experiments were performed. Horizontal lines and error bars mark median number of cells with interquartile range. Statistical analysis was performed using a Mann-Whitney U test (* $p \leq 0.05$, *** $p \leq 0.001$).

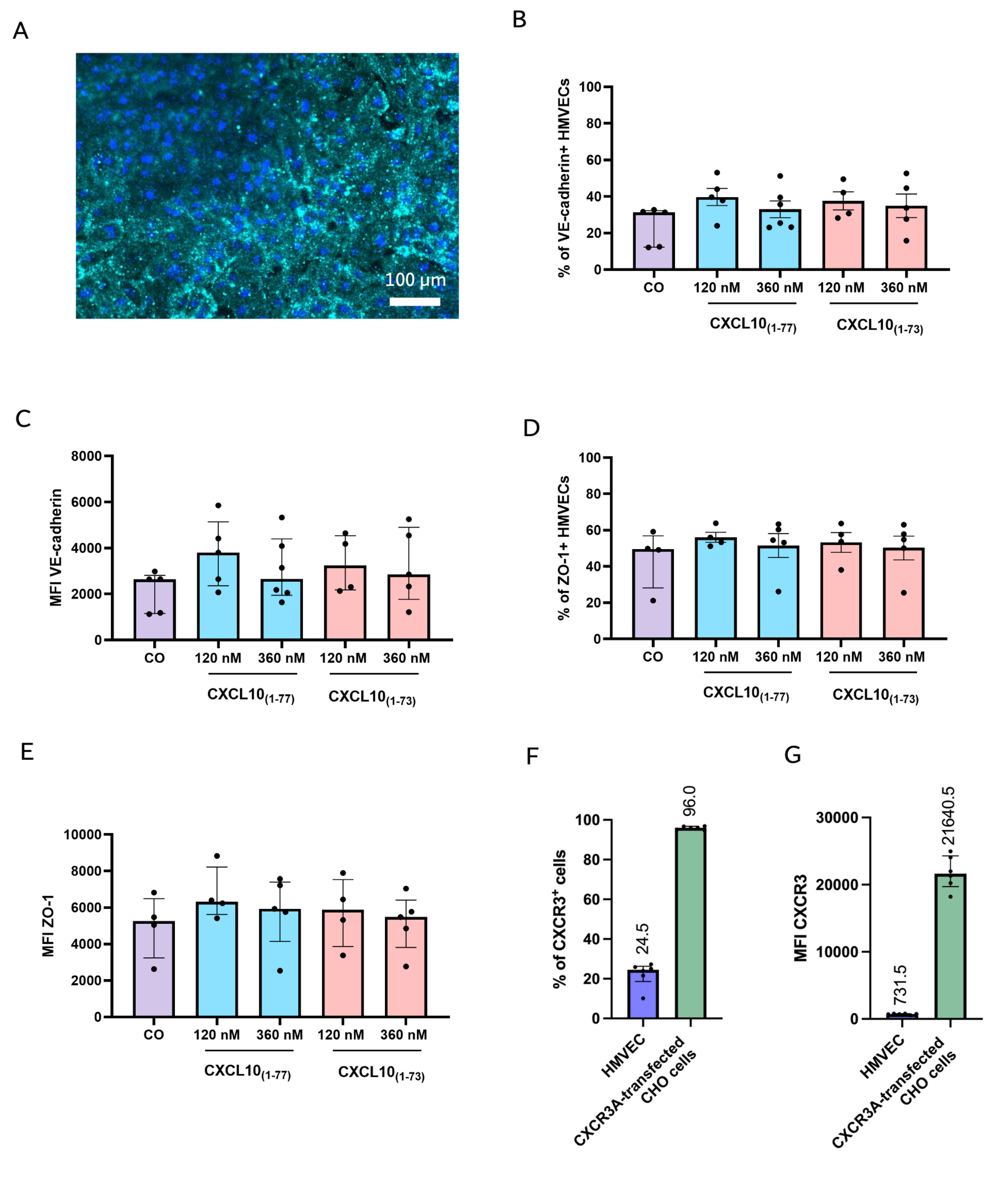

SUPPLEMENTARY FIGURE S9. VE-cadherin and ZO-1 expression by HMVEC monolayers and CXCR3 expression by HMVECs and CXCR3A-transfected CHO cells. VE-cadherin and ZO-1 expression by HMVEC monolayers upon exposure to intact $CXCL10_{(1-77)}$ and C-terminally truncated $CXCL10_{(1-73)}$ was assessed. (**A**) Confluence of the monolayer in the transwell inserts was assessed via confocal microscopy. A representative image is shown (F-actin [phalloidin] in green, cell nuclei [Hoechst] in blue) at t = 0h (before stimulation). Expression and median fluorescence intensity (MFI) of (**B**-**C**) VE-cadherin/CD144 and (**D**-**E**) ZO-1 on HMVEC (gated as $CD31^+$ cells) was evaluated through flow cytometry upon stimulation for 48 h at 37°C and 5% CO_2 with control medium EBM-2 + 3% FCS (CO) or $CXCL10_{(1-77)}$ or $CXCL10_{(1-73)}$ at the indicated doses. The data are displayed as median (± IQR) of 4 to 6 independent experiments. Mann-Whitney U test was performed (no significant differences detected). CXCR3 expression on HMVEC and CXCR3A-transfected CHO cells in culture was evaluated through flow cytometry with (**F**) proportions of CXCR3+ cells and (**G**) MFI of CXCR3. Data are shown as median (± IQR) (n = 6).

SUPPLEMENTARY TABLE S1. Overview of the antibodies used for evaluation of CXCR3 expression on PHA- and IL-2-stimulated T lymphocytes through flow cytometry.

Human Antigen	Clone	Fluorochrome	Host species	Company
CD3	UCHT1	BV785	Mouse	Biolegend
CD19	HIB19	APC-R700	Mouse	BD Biosciences
CD56	NCAM16.2	BV421	Mouse	BD Biosciences
CD26	M-A261	FITC	Mouse	BD Biosciences
CD183 (CXCR3)	1C6	PE-Cyanine 5	Mouse	BD Biosciences

SUPPLEMENTARY TABLE S2. Overview of the antibodies used for mice experiments.

Mouse Antigen	Clone	Fluorochrome	Host species	Company
CD19	1D3	FITC	Rat	BD Biosciences
CD8	53-6.7	PerCP-Cyanine 5.5	Rat	eBioscience, Invitrogen
NK1.1	PK136	PE-Cyanine7	Mouse	eBioscience, Invitrogen
CD183 (CXCR3)	CXCR3-173	PE	Hamster	Biolegend
CD4	RM4-5	APC-eFluor780	Rat	eBioscience, Invitrogen
CD45	30-F11	APC	Rat	eBioscience, Invitrogen
CD3	145-2C11	BUV395	Hamster	BD Biosciences

SUPPLEMENTARY TABLE S3. Overview of the antibodies and flow cytometry panels used for evaluation of lymphocyte adhesion molecules on HMVEC

Human Antigen	Clone	Fluorochrome	Host species	Company
CD54 (ICAM-1)	HA58	BV711	Mouse	BD Biosciences
CD31 (PECAM-1)	WM59	PE	Mouse	Biolegend
CD106 (VCAM-1)	STA	APC	Mouse	Biolegend
CD144 (VE-cadherin)	55-7H1	BV786	Mouse	BD Biosciences
ZO-1*	ZO1-1A12	FITC	Mouse	eBioscience, Invitrogen

*ZO-1 was intracellularly stained

SUPPLEMENTAL EXPERIMENTAL PROCEDURES

Cell cultures and reagents

CXCR3A-CHO cells, HMVEC, and primary T lymphocytes were all grown at 5% CO_2 and 37°C. For the use of primary T lymphocytes, Ethics Committee Research UZ/KU Leuven approved the experiments using human leukocytes (**S58418**). Human recombinant $CXCL10_{(1-77)}$ and human recombinant $CXCL12_{(1-68)}$ were purchased from PeproTech (Rocky Hill, NJ, USA), and human recombinant basic fibroblast growth factor (FGF-2) was bought from R&D Systems (Minneapolis, CA, USA).

Chemical synthesis, purification and folding of C-terminally truncated human $CXCL10_{(1-73)}$

H-Ser(OtBu)-HMPB NovaPEG resin (loading 0.59 mmol/g; 0.1 mmol; Novabiochem, Sigma-Aldrich; Saint Louis, MO, USA) was used as solid phase peptide support. Fmoc deprotection of the resin-coupled amino acid was performed via two treatments of 5 min with 20% (*v/v*) piperidine (Biosolve, Valkenswaard, the Netherlands) in N-Methyl-2-pyrrolidone (NMP; Biosolve) [2]. Deprotection was automatically repeated in case the Fmoc group removal was inefficient as determined from its UV absorption in the piperidine solution that is removed from the reaction vessel. Prior to coupling, resin particles were washed in NMP. Subsequently, O-(1H-6-chlorobenzotriazole-1-yl)-1,1,3,3-tetramethyluronium hexafluorophosphate (HCTU; 0.5 M; Iris Biotech, Marktredwitz, Germany) in 2.0 ml N,N-dimethylformamide (DMF; Acros Organics, Geel, Belgium) was added to activate 1 mmol Fmoc-protected amino acid (0.1 M), followed by the addition of 4-methylmorpholine (NMM; 1 M; Sigma-Aldrich) in DMF (1.0 ml) to improve amide coupling reaction efficiency. The HCTU-activated amino acid was transferred to the reaction vessel to enable coupling of the transferred amino acid. The generated solution was mixed and allowed to incubate at room temperature (RT) for 30 min on the resin under nitrogen atmosphere. Thereafter, resin particles were washed in NMP (3.0 ml × 4). For all residues, double coupling was executed with an additional prolongation of the coupling time for the incorporation of proline residues (Pro^{2}, Pro^{18}, Pro^{21}, Pro^{37}, Pro^{56}). Special building blocks were incorporated at key positions including the pseudoproline dipeptides Fmoc-L-Arg-L-Thr[$\Psi^{Me,Me}$Pro] (Iris Biotech) at Arg^{5}-Thr^{6}, Fmoc-L-Ile-L-Ser[$\Psi^{Me,Me}$Pro] (Iris Biotech) at Ile^{12}-Ser^{13}, Fmoc-L-Ala-L-Thr[$\Psi^{Me,Me}$Pro] (Iris Biotech) at Ala^{43}-Thr^{44} and Fmoc-L-Val-L-Ser[$\Psi^{Me,Me}$Pro] (Iris Biotech) at Val^{68}-Ser^{69} and the dipeptide building block Fmoc-L-Ile-L-Pro (Iris Biotech) at Ile^{30}-Pro^{31}. Subsequently, capping of the remaining free amino groups on the resin-bound amino acids was performed with an acetic anhydride solution (3.0 ml) containing acetic anhydride (0.5 M; Honeywell Fluka, Seelze, Germany), di-isopropylethylamine (0.129 M; DIEA; Carl Roth) and 1-hydroxybenzotriazole (0.015 M; HOBt; Acros Organics) in NMP. Finally, resin particles were washed in NMP (3.0 ml × 5). These deprotection-couple-capping cycles were reiterated until the peptide chain was completed.

For cleavage of the proline-like oxazolidine structures of the pseudoprolines and for removal of the resin and the side-chain protection groups, the synthetic material was incubated with a mixture of trifluoroacetic acid (TFA; Biosolve)/thioanisole (Acros Organics)/1,2-ethanedithiol (Honeywell Fluka)/ ultrapure water (87.5/5.0/2.5/5.0 *v/v*) and 7.5% (*w/v*) crystalline phenol (Merck, Darmstadt, Germany) at RT under continuous shaking for 2 h. The acid-labile oxazolidine of the pseudoprolines is cleaved by TFA, enabling the conversion into the Ser/Thr-containing

dipeptides [3]. Subsequently, the mixture was filtered through a Biospin filter (Bio-Rad, Temse, Belgium) to remove resin particles and proteins were precipitated and washed in cold diethyl ether (Fisher Scientific, Loughborough, UK). Afterwards, proteins were dissolved in ultrapure water, lyophilized, reconstituted in 0.1% (*v/v*) TFA in ultrapure water and purified by reverse phase high performance liquid chromatography (RP-HPLC; Waters 600 HPLC system: controller and solvent delivery system; Milford, MS, USA) using a C_4 column (10.0 × 150.0 mm, 5 μm; Higgins Analytical Inc., California, USA) and a 40-min gradient (4.0 ml/min) from 0.0 % to 80.0% (*v/v*) acetonitrile in 0.1% (*v/v*) TFA solution. The relative molecular mass (Mr) of the protein was detected by online ion trap mass spectrometry (Amazon SL, Bruker Daltonics, Bremen, Germany).

To avoid loss of peptides through adsorption to glass and plastics, 0.01% (*v/v*) 4-nitrophenyl β-D-glucuronide was added to the fractions containing purified linear protein, after which the fractions were partially dried in vacuo to obtain a confined volume of 1 ml. Subsequently, proteins were folded in a mixture —containing guanidine hydrochloride (1.0 M; Sigma-Aldrich) and tris(hydroxymethyl)-aminomethane (Tris; 0.1 M; Sigma-Aldrich) at pH 8.5— in a 2:1 ratio (buffer:protein-containing fraction) and stirred in air for 24 h [4]. Afterwards, the folded material was acidified to a pH < 4 with TFA and purified by RP-HPLC (C_8-Aquapore RP-200 column, 2.1 × 220.0 mm; PerkinElmer, Massachusetts, USA) using a 98-min gradient (0.4 ml/min) from 0.0 % to 80.0 % (*v/v*) acetonitrile in 0.1% (*v/v*) TFA solution. Automated Edman degradation (PPSQ-51A protein sequencer, Shimadzu, Kyoto, Japan) and ion trap mass spectrometry (Amazon SL, Bruker Daltonics) were used to confirm protein purity and concentration.

Biotinylation of glycosaminoglycans

For utilization in downstream surface plasmon resonance (SPR) experiments, heparin (Iduron, Alderley Edge, Cheshire, UK), heparan sulfate (HS; Iduron), and chondroitin sulfate (CS)-A sodium salt (Sigma-Aldrich) were biotinylated, as previously described [5]. To obtain oxidized GAGs (i.e., conversion of COOH-groups in aldehyde-groups), heparin, HS and CS-A were incubated with 10 mM sodium periodate in the dark in ultrapure water at RT for 30 minutes. After exchange of the buffer to a sodium acetate buffer (0.1 M, pH 5.7) using a Sephadex G-25 column (Sigma-Aldrich), the components were concentrated through ultracentrifugation in a Centricon centrifugal filter with a pore size of 3 kDa (Merck). Biotin hydrazide (5 mM in DMSO; Sigma-Aldrich) was incubated with the oxidized GAGs at RT for 2 hours, after which the suspension was again subjected to gel filtration using a Sephadex G-25 column prewashed with phosphate-buffered saline (PBS) to eliminate remnant free hydrazide molecules. Finally, successful biotinylation was corroborated by testing the biotinylated GAGs using plates pre-coated with streptavidin and streptavidin-horseradish peroxidase (HRP)-based detection of bound biotinylated GAGs in an ELISA-like assay (data not shown).

Surface plasmon resonance

Real-time binding kinetics of CXCL10 proteoforms with different GAGs were examined through SPR on a BIAcore T200 instrument (Cytiva, Uppsala, Sweden) in a similar experimental set-up as previously described [5]. Firstly, a CM4 Biosensor chip (Cytiva) was primed in running buffer (10 mM HEPES, 150 mM NaCl, 3 mM

ethylenediaminetetraacetic acid [EDTA], 0.05% surfactant P20; pH 7.4; Cytiva) and washed twice in a regeneration solution (1 M NaCl, 50 mM NaOH in ultrapure water) for 1 minute. Subsequently, carboxymethyl-groups on the chip were activated through an injection of a 1:1 mixture containing N-hydroxysuccinimide (NHS; 0.05 M) and 1-ethyl-3-[3-dimethylaminopropyl]carbodiimide hydrochloride (EDC; 0.2 M) for 15 minutes and were coupled to neutravidin (20 µg/ml; Thermo Fisher Scientific) dissolved in acetate buffer (10 mM sodium acetate; pH 5.5; Cytiva) until saturation was reached. Thereafter, the remaining activated carboxymethyl-groups that were not bound to neutravidin were quenched with 35 µl of 1 M ethanolamine hydrochloride (Cytiva; adjusted to pH 8.5 with NaOH) for 15 minutes at 10 µl/minute. Following washing in an alternative regeneration solution (1 M NaCl, 10 mM NaOH) for 1 minute, biotinylated glycosaminoglycans (GAGs; heparin, HS and CS-A) were passed over individual flow channels (10 µl/min) to attain minimal immobilization levels on a neutravidin-coated CM4 chip (Cytiva; Uppsala, Sweden; 13 response units [RU] for HS, 9 RU for CS-A, and 26 RU for heparin). For affinity determination of chemokine-GAG interactions, varying concentrations of $CXCL10_{(1-77)}$, $CXCL10_{(1-73)}$ and CXCL4 dissolved in running buffer were injected at a flow rate of 30 µl/min for 2 min. CXCL4 was included as a positive control for CS-A binding. Multiple injections of buffer were repeatedly performed between cycles for double referencing. Between cycles, extensive washing of the chip surface was performed with 1 M NaCl in ultrapure water. Responses from a reference flow cell (containing no immobilized GAG; Fc1) were subtracted from the signal of the respective GAG-bound channels to exclude non-specific interactions and alterations in refraction indexes not related to the specific chemokine-GAG interactions. Binding kinetics were analyzed with Biacore T200 Evaluation Software 3.1 (Cytiva) and fitting cycles of the experimentally-determined sensorgrams were performed by using a 1:1 binding model with mass transfer correction (maximal analyte binding capacity of the surface (Rmax) set to global; bulk refractive index contribution (RI) set to local) [6,7]. Qualitative interpretation of fitted curves on the sensorgrams and χ^2, U, t_c, SE(t_c), and residual values were used to evaluate fitting quality and mass transport limitation. The ratio of dissociation (k_{off}) and association (k_{on}) rate constants were used to estimate the dissociation equilibrium (affinity) constant (K_D). After exporting graph data from Biacore T200 Evaluation Software version 3.1, sensorgrams were created in GraphPad Prism software (version 10.0.3).

Signal transduction assays

The increase of the intracellular calcium concentration induced by CXCL10 proteoforms was evaluated on CXCR3A-transfected CHO cells [8,9]. Briefly, cells resuspended in Ham's F-12 growth medium + 10% (*v/v*) FCS were loaded with 2.5 µM of the fluorescent dye Fura-2 (Molecular Probes, Invitrogen, Carlsbad, CA) in the dark for 30 minutes at 37°C in the presence of 125 µM probenecid (ICN Biomedicals, Costa Mesa, CA, USA) and 0.01% (*w/v*) pluronic F-127 (Sigma-Aldrich). To determine autofluorescence, 5.0×10^6 cells were not loaded with Fura-2. After washing, 2.0×10^6 cells were resuspended in calcium buffer, i.e., Hanks' balanced salt solution (HBSS) supplemented with 1 mM calcium, 0.1% (*v/v*) FBS and 10 mM HEPES (pH 7.0 at 30°C through buffering with NaOH). The calcium buffer added to the Fura-2-labeled cell suspension also contained 125 µM probenecid. Upon pre-incubation of the cells at 30°C for 10 minutes and subsequent exposure to varying concentrations of chemokines, the ratio of the Fura-2 fluorescence emission at 340 and 380 nm upon excitation at 510 nm was measured in an LS50B spectrofluorimeter (PerkinElmer, Waltham, MA, USA), as previously described [9]. Afterwards, intracellular calcium values were

determined based on the incorporation of the respective fluorescence intensity ratios in the equation of Grynkiewicz *et al.* [10]. For desensitization experiments, inactive concentrations of $CXCL10_{(1-73)}$ were added prior to a stimulation with 3 nM of intact $CXCL10_{(1-77)}$. The percentage of desensitization was calculated relative to the increase of intracellular calcium in response to $CXCL10_{(1-77)}$ stimulation after the addition of calcium buffer as first stimulus [8]. To determine phosphorylation of ERK1/2 and Akt upon chemokine treatment, signal transduction was terminated by adding cold PBS and washing twice with cold PBS, whilst the plates remained on ice. Cells were lysed through addition of PBS (100 µl/well) containing 1 mM EDTA, 0.5% Triton-X-100, 5 mM NaF, 6M ureum, 1% (*v/v*) EDTA-free Halt protease inhibitor cocktail (Thermo Fisher Scientific), 1% (*v/v*) phosphatase inhibitor cocktail 2 (Sigma-Aldrich) and 1% (*v/v*) phosphatase inhibitor cocktail 3 (Sigma-Aldrich). After 15 minutes of incubation on ice, cell lysates were collected, centrifuged (8 minutes at 4°C and 490*g*), and supernatants were collected and stored at -20°C. Phosphorylated ERK1/2 (pERK1/2) and phosphorylated Akt (pAkt) induced by $CXCL10_{(1-73)}$ and $CXCL10_{(1-77)}$ relative to total protein concentration and to medium-treated cells (set to 100%) was compared. Total protein concentrations in cell supernatants were measured through the bicinchoninic acid (BCA) protein assay (Pierce, Rockford, IL, USA). The amount of pERK1/2 and pAkt in the cell supernatants was determined using an ERK1 (Thr^{202}/Tyr^{204})/ERK2(Thr^{185}/Tyr^{187}) and Akt (Akt1 [Ser^{473}], Akt2 [Ser^{474}] and Akt3 [Ser^{472}]) DuoSet enzyme-linked immunosorbent assays (ELISA) (R&D Systems).

Multiscreen chemotaxis assay with primary T lymphocytes

Pre-coating of the 96-well filter plates was performed with 20 µg/ml bovine plasma fibronectin (FN; Gibco; Thermo Fisher Scientific; cat#33010-018, lot#2498287), 20 µg/ml human plasma FN (Corning, New York, USA; cat#354008, lot#3016002) or 10 µg/ml type I human collagen (Sigma-Aldrich; cat#CC050, lot#3856637) in Dulbecco's Phosphate Buffered Saline [DPBS] overnight. T lymphocytes that migrated to the receiver plate were quantified via the luminescence ATP detection assay (PerkinElmer, Waltham, MA) according to the manufacturer's instruction. The ratio of the luminescence value of the wells containing chemoattractant and mean luminescence value of the buffer controls (spontaneous migration) was used to determine the chemotactic index (CI). In parallel with the multiscreen assay, CXCR3 expression on T lymphocytes stimulated with PHA- and IL-2 was evaluated through flow cytometry. For all washing steps of flow cytometry, cells were centrifuged at RT for 5 min at 300*g*. Equal volumes of PHA- and IL-2-stimulated T cells (100 µl, 5×10^6 cells/ml) were resuspended in PBS containing 100 µM sitagliptin (Januvia; Merck Sharpe & Dohme [MSD] Whitehouse Station, NJ, USA) and washed once in 1 ml PBS + 100 µM sitagliptin to remove remnant FCS. Thereafter, cells were incubated with 5 µl Human FcR block (Miltenyi Biotec, Bergisch Gladbach, Germany) and 1 µl Zombie Aqua 510 (Biolegend; San Diego, California, USA) for 15 min at RT. After washing the cells once in flow cytometry buffer (PBS + 2% [*v/v*] FCS + 2 mM [*w/v*] EDTA), cells were stained with in-house titrated antibodies (**Suppl. Table 1**) for 30 min on ice in the dark. Cells were washed once in 1 ml flow cytometry buffer and resuspended in 300 µl fixative (PBS + 0.4% [*v/v*] formaldehyde). Then, cells were immediately analyzed using a BD LSRFortessa X-20 (BD Biosciences, San Jose, California, USA) and data analysis was performed using FlowJo Software version 10.7.1.

In vitro toxicity assay

A mixture of 0.5 µM ethidium homodimer-1 (EthD-1) and 1 µM calcein-acetoxymethyl ester in DMEM FluoroBrite (Gibco) + 0.4% (*v/v*) FCS was added to all wells (100 µl/well) 1 h before read-out. Fluorescence values in the green and red channel were measured in each well using the IncuCyte S3 Live-Cell Analysis System (37°C, 5% CO2; Essen Bioscience, Newark, UK). The ratio of the area of the calcein-stained viable cells compared to the total cell area was used to calculate the percentage of cell viability using the basic analyzer unit of the IncuCyte S3 2017 Software (Essen Bioscience), as previously described [5]. A positive control for cell death was incorporated by exposing control medium-treated cells to 2% (v/v) Triton X-100 (Merck) 1 h prior to the endpoint of the experiment.

Scratch wound assay

Pictures of the 96-well plates were acquired by time-lapsed microscopy with a 10× objective every hour for 17h and processed with IncuCyte S3 2017 Software (Essen Bioscience). The Incucyte Scratch Wound Analysis Software Module uses an algorithm to generate two masks, i.e. the scratch wound mask (representing the wound width) and the confluence mask (representing the cell confluence of the wound region). An initial scratch wound mask defines the initial wound and its wound boundaries at 0h. For each time-lapsed microscopy picture, the scratch wound mask and confluence mask is computed. Using these masks, the wound confluence and relative wound density were calculated. Wound confluence refers to the fractional area of the wound that is occupied by cells and reflects solely cell migration, whereas the relative wound density reflects cell migration and invasion. The relative wound density represents the density of the wound region relative to the density of the cell region and accounts for background density of the wound at the initial time point (**Eq. 1**). To compare wound closure after incubation with $CXCL10_{(1-73)}$ and $CXCL10_{(1-77)}$ relative to control medium or FGF-2-treated cells, wound confluence and relative wound density measured upon incubation with control medium or FGF-2 were set to 100%. To acquire additional high-resolution images, the wound area was stained with 1 µM calcein-acetoxymethyl ester in EMB-2 + 1% (v/v) FCS and imaged at 0 h and 17 h through a 10× objective inverted Axiovert 200M microscope (Carl Zeiss Microscopy GmbH, Oberkochen, Germany).

$$\%\ \text{relative wound density}\ (t)\ = 100\ \times\ \frac{(w[t]-w[0])}{(c[t]-w[0])} \qquad (1)$$

EQUATION 1 | The relative wound density at a specific time point 't' represents the density of the wound region (w) at that time 't' relative to the density of the cell region (c) at time 't' and accounts for background density of the wound at the initial time point (t = 0).

In vivo cell migration assay

For flow cytometry, equal volumes of isolated peritoneal cells (100 µl, 5 × 10^6 cells/ml) were washed once in 500 µl flow cytometry buffer, stained and analysed. Subsequently, cells were treated with 0.5 µl Mouse FcR block (Miltenyi Biotec) and 1 µl Zombie Aqua 510 (Biolegend) for 15 min at RT, followed by washing the cells once in 1 ml flow cytometry buffer. Thereafter, cells were stained with in-house titrated antibodies (**Suppl. Table 2**),

washed, fixed and analyzed (*vide supra)*. The Ethics Committee of KU Leuven approved the experiments (**P198/2017**).

CD26 activity assay

Absorbance of *p*-nitroanilide substrate was monitored at 405 nm using a spectrophotometer (BioTek, PowerWave XS, Winooski, VT, USA) at 37°C every 5 min for 14 h. OD values reflect the kinetics of CD26-mediated enzymatic conversion and were used to determine CD26 enzymatic activity. Using the Lambert-Beer law, the slopes of the CD26 activity curves — representing the amount of converted substrate per minute — were used to calculate the CD26 enzymatic activity (**Eq. 2**). Sitagliptin (100 µg/ml; Januvia) was used to confirm CD26 specificity.

$$\text{Enzymatic activity (U/l)} = \frac{\Delta C}{\Delta t} = \frac{\Delta A}{\Delta t \times \varepsilon \times l} \qquad (2)$$

EQUATION 2 | The computed CD26 enzymatic activity is calculated as the slope of the absorbance (A) in function of the time (t) in the linear part of the graph, according to the Lambert-Beer law with the molar attenuation coefficient (ε) and the optical path length (l) [λ = 405 nm; ε of p-nitroaniline = 9500 M^{-1} cm^{-1}; l = 0.625 cm (200 μl) in a 96-well plate].

To establish a calibration curve of the soluble CD26 activity (y-axis) in function of the sitagliptin concentration (x-axis) [1], a serial dilution of sitagliptin was added to peritoneal fluids of plasma of naïve untreated mice. CD26 activity was measured for each sample of the serial dilution and expressed as a percentage compared to a naïve sample (which contained neither endogenous nor exogenously added sitagliptin) (**Fig. S7B**). The measured *in vitro* CD26 activity of each experimental sample of peritoneal fluid of mice was also expressed as a percentage compared to this naïve sample. Subsequently, the computed calibration curve based on the serial dilution of sitagliptin was used to calculate the concentration of sitagliptin (x-value) in the experimental sample based on the percentage of *in vitro* CD26 activity. The dilution factor-corrected sitagliptin concentration was then used to determine the residual CD26 activity *in vivo* using the calibration curve.

Confocal microscopy of transwell inserts of the vascular permeability assay

Transwell inserts were washed with PBS and fixed with PBS + 4% (*v/v*) formaldehyde overnight. Following washing in PBS and permeabilization with 0.1% Triton-X100, 1% (*v/v*) BSA in PBS was added for 30 min at RT to block. Upon sequential incubation with Alexa Fluor 647 Phalloidin (Thermo Fisher Scientific; Waltham, MA, USA) and Hoechst 33342 (Thermo Fisher Scientific) with washing steps with PBS in between, membranes were removed and put on a glass slide in mounting medium. Imaging was done through a 10× objective using an Andor Dragonfly Spinning Disk Confocal Microscope (Oxford Instruments, Oxfordshire, England).

Evaluation of expression of lymphocyte adhesion molecules and junctions on endothelial cells

For flow cytometry, detached cells of the 6-well plates (200 000 cells/tube) were incubated with 5 µl Human FcR block for 15 min at RT, and washed once in 1 ml flow cytometry buffer. Surface staining with in-house titrated antibodies (**Suppl. Table 3**) was performed for 25 min on ice in the dark. Following another wash-step in 1 ml flow

cytometry buffer, cells were fixed, permeabilized and washed using BD Cytofix/Cytoperm kit (BD Biosciences) according to manufacturer's instructions. Intracellular staining with anti-human zona occludens 1 (ZO-1) antibodies was performed for 25 min on ice in the dark. Then, cells were washed in 1 ml flow cytometry buffer, fixed in 300 µl fixative and analyzed (HMVEC gated as Platelet Endothelial Cell Adhesion Molecule 1 [PECAM-1/CD31]$^+$ cells).

SUPPLEMENTAL REFERENCES

1. Matheeussen V, Lambeir AM, Jungraithmayr W, Gomez N, Mc Entee K, Van der Veken P, et al. Method comparison of dipeptidyl peptidase IV activity assays and their application in biological samples containing reversible inhibitors. Clin Chim Acta. 2012;413(3–4):456–62. doi: https://doi.org/10.1016/j.cca.2011.10.031

2. Loos T, Mortier A, Proost P. Chapter 1. Isolation, identification, and production of posttranslationally modified chemokines. Methods Enzymol. 2009;461:3–29. doi: https://doi.org/10.1016/S0076-6879(09)05401-9

3. Postma TM, Albericio F. Cysteine pseudoprolines for thiol protection and peptide macrocyclization enhancement in fmoc-based solid-phase peptide synthesis. Org Lett. 2014;16(6):1772–5. doi: https://doi.org/10.1021/ol5004725

4. Clark-Lewis I, Vo L, Owen P, Anderson J. Chemical synthesis, purification, and folding of C-X-C and C-C chemokines. Vol. 287, Methods in Enzymology. 1997. p. 233–50. doi: https://doi.org/10.1016/S0076-6879(97)87018-8

5. De Zutter A, Dillemans L, Berghmans N, Noppen S, Crijns H, Verscheure P, et al. A stabilized CXCL9(74–103)-derived peptide selectively inhibits proliferation, adhesion and metastasis of tumor cells that express high levels of heparan sulfate. Int J Biol Macromol. 2022;222(Pt B):2808–22. doi: https://doi.org/10.1016/j.ijbiomac.2022.10.060

6. Li S, Pettersson US, Hoorelbeke B, Kolaczkowska E, Schelfhout K, Martens E, et al. Interference with glycosaminoglycan-chemokine interactions with a probe to alter leukocyte recruitment and inflammation In Vivo. PLoS One. 2014;9(8):e104107. doi: https://doi.org/10.1371/journal.pone.0104107

7. Dyer DP, Salanga CL, Volkman BF, Kawamura T, Handel TM. The dependence of chemokine-glycosaminoglycan interactions on chemokine oligomerization. Glycobiology. 2015;26(3):312–26. doi: https://doi.org/10.1093/glycob/cwv100

8. Proost P, Schutyser E, Menten P, Struyf S, Wuyts A, Opdenakker G, et al. Amino-terminal truncation of CXCR3 agonists impairs receptor signaling and lymphocyte chemotaxis, while preserving antiangiogenic properties. Blood. 2001;98(13):3554–61. doi: https://doi.org/10.1182/blood.V98.13.3554

9. Mortier A, Loos T, Gouwy M, Ronsse I, Van Damme J, Proost P. Posttranslational modification of the NH2-terminal region of CXCL5 by proteases or peptidylarginine deiminases (PAD) differently affects its biological activity. J Biol Chem. 2010;285(39):29750–9. doi: https://doi.org/10.1074/jbc.M110.119388

10. Grynkiewicz G, Poenie M, Tsien RY. A new generation of Ca2+ indicators with greatly improved fluorescence properties. J Biol Chem. 1985;260(6):3440–50. doi: https://doi.org/10.1016/S0021-9258(19)83641-4
